# Super-Resolution Correlative Light and Electron Microscopy (SR-CLEM) Reveals Novel Ultrastructural Insights Into Dendritic Cell Podosomes

**DOI:** 10.3389/fimmu.2018.01908

**Published:** 2018-08-22

**Authors:** Ben Joosten, Marieke Willemse, Jack Fransen, Alessandra Cambi, Koen van den Dries

**Affiliations:** ^1^Department of Cell Biology, Radboud Institute for Molecular Life Sciences, Radboud University Medical Center, Nijmegen, Netherlands; ^2^Microscopic Imaging Center, Radboud Institute for Molecular Life Sciences, Radboud University Medical Center, Nijmegen, Netherlands

**Keywords:** super-resolution microscopy, scanning electron microscopy, dendritic cells, podosomes, actin, vinculin, zyxin

## Abstract

Podosomes are multimolecular cytoskeletal structures that coordinate the migration of tissue-resident dendritic cells (DCs). They consist of a protrusive actin-rich core and an adhesive integrin-rich ring that contains adaptor proteins such as vinculin and zyxin. Individual podosomes are typically interconnected by a dense network of actin filaments giving rise to large podosome clusters. The actin density in podosome clusters complicates the analysis of podosomes by light microscopy alone. Here, we present an optimized procedure for performing super-resolution correlative light and electron microscopy (SR-CLEM) to study the organization of multiple proteins with respect to actin in podosome clusters at the ventral plasma membrane of DCs. We demonstrate that our procedure is suited to correlate at least three colors in super-resolution Airyscan microscopy with scanning electron microscopy (SEM). Using this procedure, we first reveal an intriguing complexity in the organization of ventral and radiating actin filaments in clusters formed by DCs which was not properly detected before by light microscopy alone. Next, we demonstrate a differential organization of vinculin and zyxin with respect to the actin filaments at podosomes. While vinculin mostly resides at sites where the actin filaments connect to the cell membrane, zyxin is primarily associated with filaments close to and on top of the core. Finally, we reveal a novel actin-based structure with SEM that connects closely associated podosome cores and which may be important for podosome topography sensing. Interestingly, these interpodosomal connections, in contrast to the radiating and ventral actin filaments appear to be insensitive to inhibition of actin polymerization suggesting that these pools of actin are not dynamically coupled. Together, our work demonstrates the power of correlating different imaging modalities for studying multimolecular cellular structures and could potentially be further exploited to study processes at the ventral plasma membrane of immune cells such as clathrin-mediated endocytosis or immune synapse formation.

## Introduction

Correlative light and electron microscopy (CLEM) bridges the gap between light microscopy (LM) and electron microscopy (EM). With CLEM, the EM adds structural and cellular context to the LM images while the LM provides a specific molecular context to the EM images, thereby offering unique and complementary information from the same sample (1). CLEM data obtained with EM (lateral resolution <2 nm) and diffraction limited LM (lateral resolution ~250 nm) are usually hard to interpret since the detailed structures from the EM are not easily identified in the LM images, something which is often referred to as the resolution gap. The developments of various super-resolution (SR) LM techniques over the past decades have greatly improved the lateral resolution of LM to ~140 nm–~10 nm depending on the technique of choice (2), thus creating new opportunities for correlating SR-LM with EM (SR-CLEM) (3, 4).

Podosomes are cytoskeletal structures used by osteoclasts to create a tight sealing zone to facilitate bone resorption and by innate immune cells such as macrophages and dendritic cells (DCs) to cross basement membranes and slowly migrate through peripheral tissues (5, 6). Moreover, podosomes have been shown to be involved in the uptake of foreign antigens by DCs (7). Podosomes consist of an actin-rich protrusive core that is surrounded by an integrin-rich adhesive ring. Typically, hundreds of podosomes are grouped into large clusters, where individual podosomes are interconnected by radiating actin filaments (8). Importantly, the detailed actin organization of podosomes is difficult to study by LM alone, since podosome cores are small (~700 nm), actin dense structures and the interconnecting actin filaments are only resolved by super-resolution microscopy (8). Therefore, scanning EM (SEM) of ventral plasma membranes, where podosomes are assembled, has been applied before to study the ultrastructural organization of podosomes in osteoclasts (9–11). Although these studies provided invaluable insight into the ultrastructural organization of podosomes, the lack of information on the nanoscale organization of specific proteins makes interpretation of the SEM images alone challenging. The individual limitations of LM and SEM highlight the need for correlative imaging approaches to study the podosome ultrastructure.

Podosomes contain dozens of proteins that are either primarily associated with the actin core or the integrin ring where also the radiating actin filaments are located. Vinculin and zyxin are two cytoskeletal adaptor proteins involved in cell adhesion and classically associated with the ring (12). The function, however, of vinculin and zyxin is very different with vinculin being directly involved in connecting actin to integrins (13), while zyxin may be more involved in the stress fiber repair (14), suggesting that their localization may also be different. Interestingly, by averaging the fluorescent signal from hundreds of podosomes in diffraction limited microscopy, we have shown before that zyxin resides more close to the core than vinculin (15). Moreover, it has been shown by SR-LM for focal adhesions that vinculin and zyxin reside in different vertically separated regulatory layers (16). Studying the exact mutual localization of multiple proteins with respect to actin structures in podosomes is instrumental for our understanding of how the dozens of functionally different proteins together regulate podosome function and requires a procedure that combines multicolor SR-LM microscopy with the ultrastructural actin organization as imaged by SEM.

Airyscan imaging is a relatively new laser scanning imaging technique that is based on a concentrically arranged hexagonal detector array, and achieves nearly twice the resolution (lateral resolution ~140 nm) in all three dimensions compared to conventional confocal laser scanning microscopy. Compatible with virtually all conventional fluorophores, Airyscan imaging allows simultaneous labeling of multiple proteins and Airyscan SR-CLEM therefore offers new opportunities to correlate the SR-LM information from multiple fluorescently labeled cellular constituents with the ultrastructural organization imaged by SEM. This prompted us to develop a novel imaging pipeline to correlate these imaging modalities for the detailed investigation of the mutual organization of actin, vinculin, and zyxin in podosomes.

Here, we present a novel SR-CLEM imaging pipeline that enables the sequential imaging of multicolor samples using Airyscan SR-LM imaging followed by SEM image acquisition. We present experimental results confirming the feasibility of our SR-CLEM procedure to obtain an optimal overlay of the fluorescence information of multiple podosome proteins with the ultrastructural information obtained by SEM across entire cells. Combining the two imaging modalities allowed us to precisely determine the mutual localization of vinculin and zyxin in DC podosomes. Also, we identify a novel actin-rich structure that connects closely associated podosome cores on flat surfaces as well as on topographical cues and we have investigated the effects of inhibition of actin polymerization on these different actin structures. We foresee that the relatively straightforward procedure that we developed can also be applied to study various other multi-molecular structures at the ventral plasma membrane such as clathrin-coated pits and the immune synapse.

## Materials and methods

### Airyscan and SEM correlative imaging pipeline

**Figure 1:**
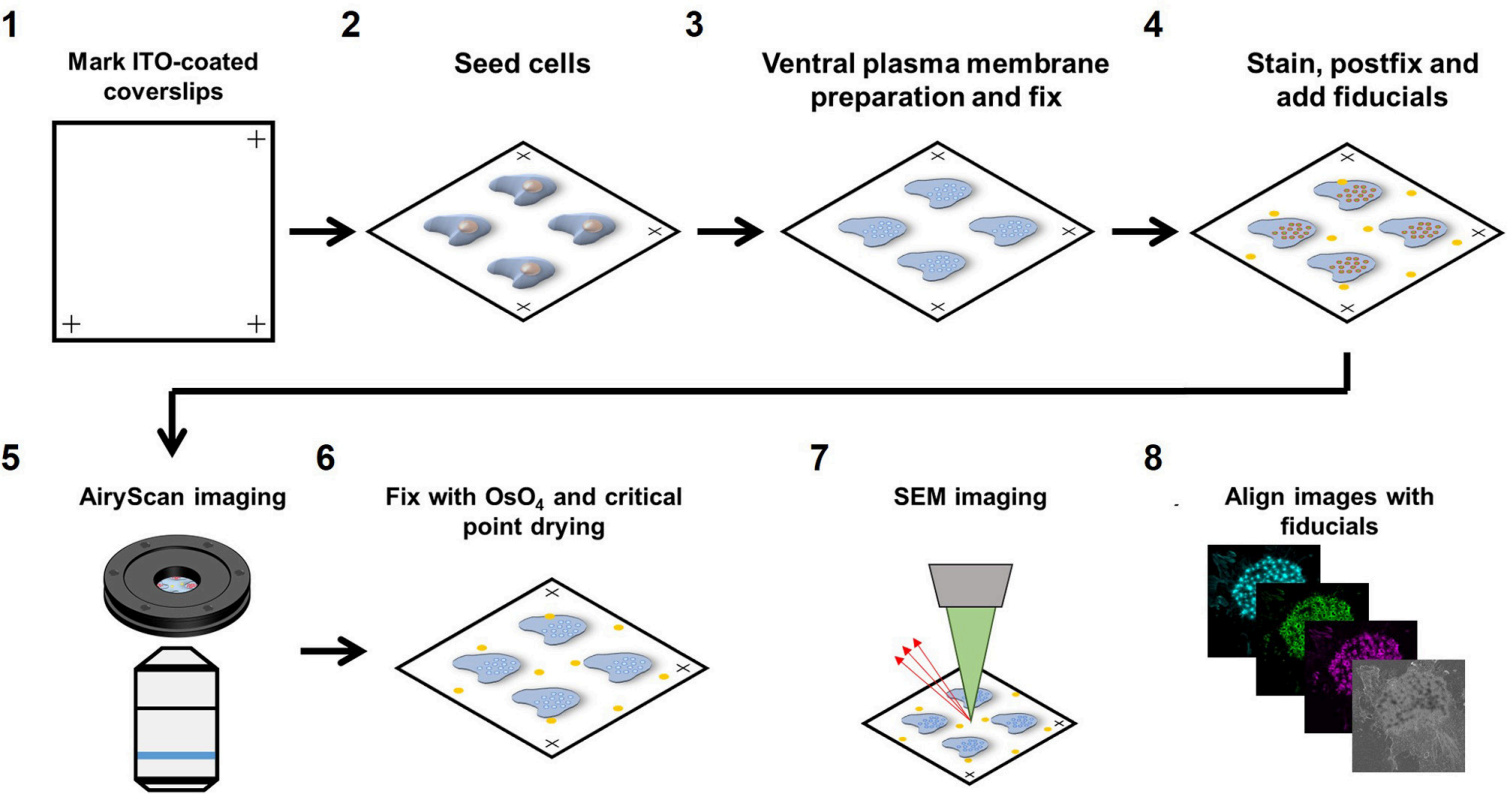
Airyscan and SEM correlative imaging pipeline. **(1)** ITO-coated coverslips are marked on three edges for stage calibration on the Zeiss LSM880 and Sigma 300. **(2)** Cells are seeded on the marked coverslips for at least 3 h. **(3)** Live cells are sonicated to prepare VPMs and immediately fixed. **(4)** Sample is immunolabeled for the proteins of interest, fixed for the second time and fiducials are added. **(5)** The sample is imaged with Airyscan microscopy. **(6)** The sample is dehydrated, critical point dried and then sputtered with 5 nm chromium. **(7)** The sample is imaged with SEM microscopy. **(8)** Fiducials are used to align the LM and SEM image using Matlab.

We developed a novel workflow to correlate SEM with multi-color super-resolution Airyscan. Briefly, samples were first prepared for Airyscan imaging under wet conditions (PBS) after which the sample was dehydrated with ethanol followed by critical point drying to perform SEM imaging. Below, we provide a step-by-step detailed protocol for sample preparation (Figure 1).

#### Step 1. mark indium-tin-oxide (ITO) coated coverslips

22 × 22 mm ITO coated coverslips were marked in three corners with an engraving pen with diamond tip. Marking the corners of the coverslips allowed calibration of the motorized stages on both the Zeiss LSM880 confocal microscope with Airyscan detector (Carl Zeiss AG, Jena, Germany) as well as the Zeiss Sigma 300 Scanning Electron Microscope (Carl Zeiss AG, Jena, Germany), saving time and effort in finding back the same cells on both instruments. After marking, coverslips were washed in 100% ethanol for sterilization and washed three times in PBS before seeding the cells.

#### Step 2. seed cells

Marked coverslips were placed in a 6 well plate and 0.2^*^10^6^ cells/well were added to the well and left to adhere to the coverslips for 3 h in RPMI medium supplemented with 10% FCS before preparing the ventral plasma membranes (VPM). For DCs, 3 h is sufficient for strong cell adhesion to allow VPM preparation but other cell types may need a longer adherence time. To study the role of actin polymerization, cells were stimulated with 2.5 μg/ml cytochalasin D (CytoD) for 10 min prior to VPM preparation.

#### Step 3. ventral plasma membrane preparation

To prepare VPMs, cells were briefly sonicated. Sonication was performed using a Sartorius Labsonic P sonicator with cycle set at 1 and amplitude at 20% output. First, the sonicator tip was placed in a glass beaker containing 100 ml prewarmed hypotonic PHEM buffer (20% PHEM: 6 mM PIPES, 5 mM HEPES, 0.4 mM Mg_2_SO_4_, 2 mM EGTA). Next, coverslips were taken from the 6 well plate and held 1–2 cm below the sonicator tip at a 45 degrees angle in the hypotonic PHEM solution. Cells were sonicated for ~1 s and directly after sonication, coverslips were transferred to a 6 well plate containing a prewarmed (37°C) PBS solution with 4% paraformaldehyde and 0.05% glutaraldehyde and incubated for 30 min at room temperature. VPM efficiency was about 50% within the area that was closest to the tip (Supplementary Figure 1A).

#### Step 4. stain, fix and add fiducials

For staining, samples were first blocked for 60 min with PBS containing 20 mM glycine and subsequently incubated with the appropriate primary antibodies for 60 min. Samples were subsequently incubated for 60 min with the appropriate fluorescently labeled secondary antibodies together with fluorescently labeled phalloidin to visualize the actin cytoskeleton. Since Airyscan imaging is performed under wet conditions, conventional fluorophores could be used for protein visualization (Alexa488, Alexa568, and Alexa647). After staining, samples were post-fixed with 2% paraformaldehyde and fiducials were added (1:2,000 diluted 0.2 μm Tetraspeck beads). Tetraspeck beads are visible by both SEM and LM and can therefore be used as fiducials for precise image alignment after image acquisition.

#### Step 5. airyscan imaging

Prior to Airyscan imaging, coverslips were mounted into a self-designed low drift magnetic imaging chamber. First, the three marks at the edges of the coverslip were located and the coordinates of the marks were stored in the shuttle and find option in the Zeiss ZEN software. Images were obtained at room temperature with a 63 × Plan Apochromat (1.4 NA) oil objective on the Airyscan array detector and processed using the Airyscan processing toolbox in the ZEN software. Comparing confocal with Airyscan laser scanning microscopy demonstrated the 1.7 increased resolution and the ability of Airyscan imaging to properly resolve diffraction limited actin filaments (Supplementary Figure 2) Only cells with a sufficient number of Tetraspeck beads (>= 3) in the field of view and good VPM preservation were selected for correlative imaging.

#### Step 6. fix with OsO_4_ and critical point drying (CPD)

After Airyscan imaging, samples were postfixed with 1% OsO_4_ in 0.1 M phosphate buffer for 10 min, and washed in MQ. Samples were subsequently dehydrated in a graded ethanol series before critical point drying (Polaron E3000, Quorum Technologies Ltd., East Sussex, UK). Samples were finally sputtered with 5 nm chromium (Quorum Q150TS, Quorum Technologies Ltd., East Sussex, UK) before SEM imaging.

#### Step 7. SEM imaging

SEM imaging was performed in a Zeiss Sigma 300 microscope (Carl Zeiss AG, Jena, Germany) equipped with the ATLAS 5 external scanner and software (Fibics, Canada) and the shuttle and find option in the Zeiss ZEN Blue software. The coordinates of the marks were recorded allowing the retrieval of the same field of view as acquired in the LSM880 Airyscan. With the SEM, unroofed cells were easily identified (Supplementary Figure 1B). SEM images were acquired at 3–5 kV, with a 30 nm aperture and a working distance of 7 mm using the InLens detector. Brightness and contrast were set at ~50 and ~30%, respectively. Large field of view images were recorded using the Zeiss Atlas software with a pixel resolution of 2 nm.

#### Step 8. align images using fiducials

After image acquisition, the immunofluorescence and SEM images were aligned using the Tetraspeck beads in the field of view. The center of mass of the fiducials was collected in the green fluorescence channel as well as the SEM image with ImageJ. Since the sample is distorted minimally from the LM to the SEM, we initially reasoned that a combination of scaling, translation and rotation would be sufficient to align the SEM and LM image. This, however, resulted in a poor image alignment and additional shearing was essential for proper image alignment (Supplementary Figure 1C). For image alignment, the *cpt2tform* and *imtransform* functions in Matlab were used to apply a 2D affine spatial transformation to the SEM image. Confirmation of alignment accuracy was done by post-alignment center of mass determination of fiducials and the mean alignment error was <10 nm. Alignment between the three fluorescence channels was not necessary as the beads already showed a near to perfect overlap between these channels.

### Merging LM-SEM images

After image alignment, images were prepared for LM-SEM merge using Fiji. First, LM images were thresholded with a local threshold with radius 5 (mean for actin images and phansalkar for vinculin and zyxin images). The resulting mask values of 255 were set to 90 and a Gaussian blur filter with radius 10 was applied. Appropriate lookup tables were applied (cyan, green and magenta) and the resulting images were merged with the SEM image.

### Preparation of human DCs

DCs were generated from monocytes, which were isolated from peripheral blood mononuclear cells as described previously (17, 18). Monocytes were derived either from buffy coats or leukapheresis products, purchased at Sanquin blood bank, Nijmegen, the Netherlands. Plastic-adherent monocytes were cultured for 6 days in RPMI 1640 medium (Life Technologies, Carlsbad, CA, USA) supplemented with 10% fetal bovine serum (Greiner Bio-One, Kremsmünster, Austria), 1 mM Ultraglutamine (BioWhittaker, Inc., Walkersville, MD, USA), antibiotics (100 U/mL penicillin, 100 μg/mL streptomycin, and 0.25 μg/mL amphotericin B; Gibco, Grand Island, NY, USA), IL-4 (300 U/mL), and GM-CSF (450 U/mL) in a humidified, 5% CO_2_ containing atmosphere.

### Antibodies and materials

The following antibodies were used: mouse anti-vinculin (Sigma-Aldrich) and goat anti-zyxin (Santa Cruz Biotechnology, Inc., Santa Cruz, CA, USA). Alexa488-conjugated phalloidin (Invitrogen Corporation, Carlsbad, CA, USA) was used to stain F-actin. Cytochalasin D was purchased from Sigma-Aldrich. ITO coated coverslips were purchased from SPI supplies (West Chester, PA, USA) and were rubbed (3 strokes) over P400 grit size sandpaper to generate topographical cues. 0.2 μm Tetraspeck beads were purchased from Invitrogen (Carlsbad, CA, USA).

### Fluorescence profile analysis

To quantify the localization vinculin, zyxin and actin in podosomes as a function of x, y, and z, we used a semi-automatic self-developed ImageJ macro that (1) recognizes the podosome core centers based on the actin image, (2) draws a vertical line of ~3 μm through the center of the core that rotates around its center (36 steps of 10 degrees) and collects an orthogonal view for every line, and (3) produces an average radial orthogonal view for each podosome core. We used these radial orthogonal views to retrieve the fluorescence profiles as a function of x, y, and z. For the x, y profiles, horizontal lines were drawn through the orthogonal views and intensity profiles were taken at the four corresponding z-sections for each channel for each podosome. For the z profiles, a diagonal line was drawn through the orthogonal views at a distance of 200 nm (zyxin), 360 nm (vinculin), 0 nm (core actin) and 1,000 nm (network actin) from the podosome core center. All profiles were normalized to the minimum and maximum (of all z-sections) for visualization and comparison. For the CytoD fluorescence intensity profiles as a function of the distance from the podosome center, the values were normalized to the control values on VPMs for comparison. All ImageJ macro codes used for the analyses in this manuscript are available upon request.

## Results

### Ventral plasma membrane (VPM) preparation and critical point drying (CPD) preserves podosome structure

**Figure 2:**
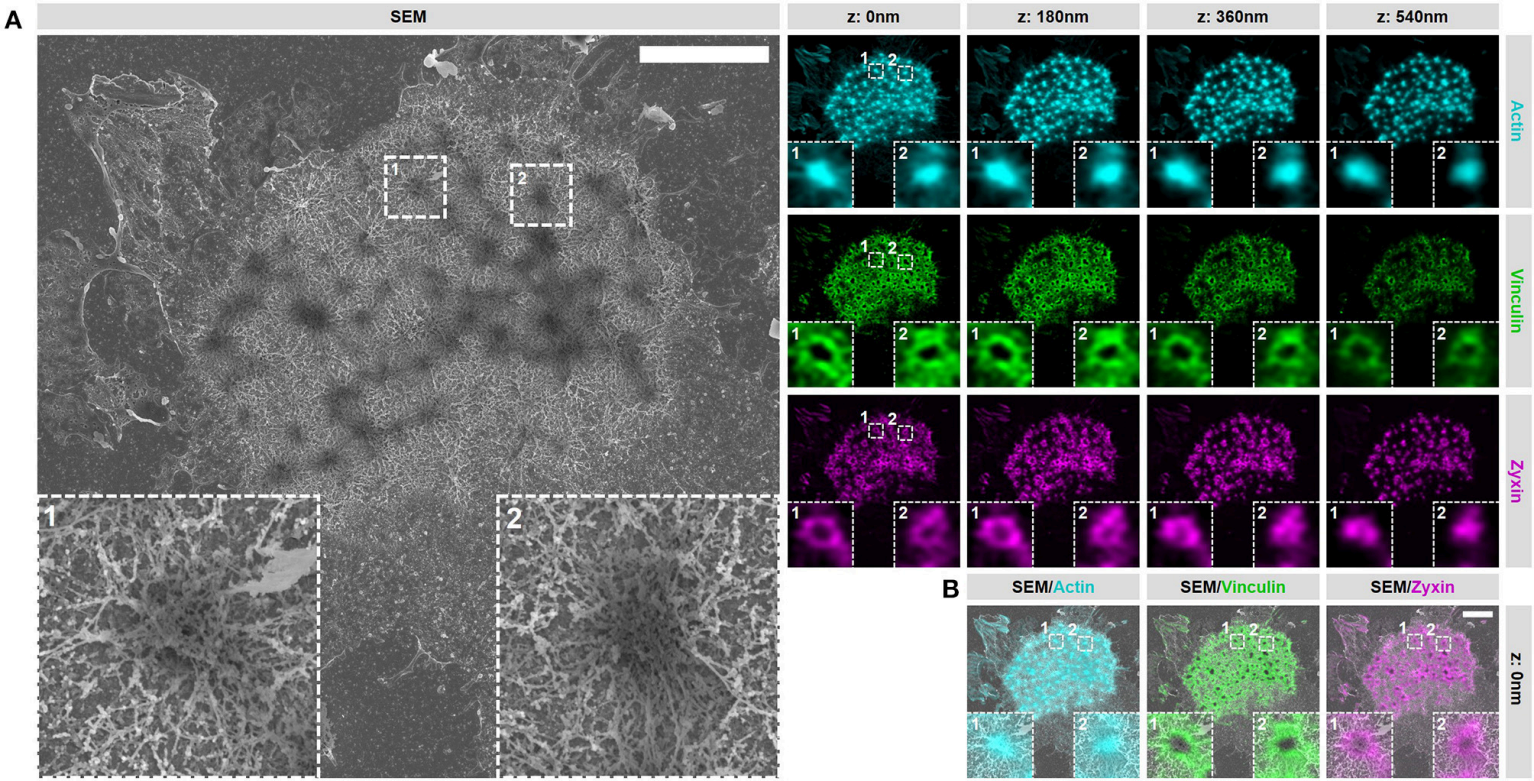
VPM preparation and CPD procedure preserve podosome organization. **(A)** DCs were seeded on glass coverslips and after VPM preparation, cells were fixed and stained for actin (cyan), vinculin (green) and zyxin (magenta). After CPD, DCs were imaged by SEM (gray). Shown are representative images of all three channels in 3 dimensions and the corresponding SEM image. Insets depict two representative podosomes within the cluster. **(B)** Shown are the SEM-LM overlays for all three channels for the same cells as in **(A)** Scale bar = 5 μm.

In order to gain access to the ultrastructure of podosomes by SEM, the cell has to be unroofed (VPM preparation) and dried by CPD, both harsh procedures that could alter the structure of cellular structures. To investigate whether cellular actin features are sufficiently preserved, we examined the organization and structure of podosomes after SR-CLEM sample preparation. For this, we first prepared VPMs and labeled them for actin to visualize the podosome core and radiating filaments, and for vinculin and zyxin to label the podosome ring. By acquiring 3D image stacks with Airyscan super-resolution microscopy, we observed that the actin cores as well as the radiating actin filaments were preserved after VPM preparation (Figure 2A). To note, Airyscan microscopy, in contrast to conventional confocal microscopy, resolves the network of diffraction limited actin filaments that have been shown to radiate from podosome cores before by SEM (9) and STORM super-resolution microscopy (8). We also found that vinculin and zyxin were still present in the podosome cluster after VPM preparation (Figure 2A). Moreover, we observed a specific localization of vinculin to the radiating actin filaments as we had observed previously with STORM super-resolution microscopy (8). We next performed CPD and imaged the VPMs by SEM. We observed that podosome clusters, individual podosomes and the associated network of radiating filaments were easily identified in the SEM image (Figure 2A). Moreover, after correlating the Airyscan and SEM images, we noted a near perfect overlay of these podosomal features, which can be observed by both imaging modalities, across the entire cell (Figure 2B).

Together, these results indicate that proteins associated with the core and ring are still present within podosome clusters on VPMs and demonstrate that the podosome structure is sufficiently preserved after VPM preparation for further investigation. Moreover, they also demonstrate that our SR-CLEM imaging pipeline allows the simultaneous visualization of at least three proteins in LM, all of which can be correlated with the ultrastructural information obtained by SEM across entire cells.

### Podosome clusters contain different types of actin filaments

**Figure 3:**
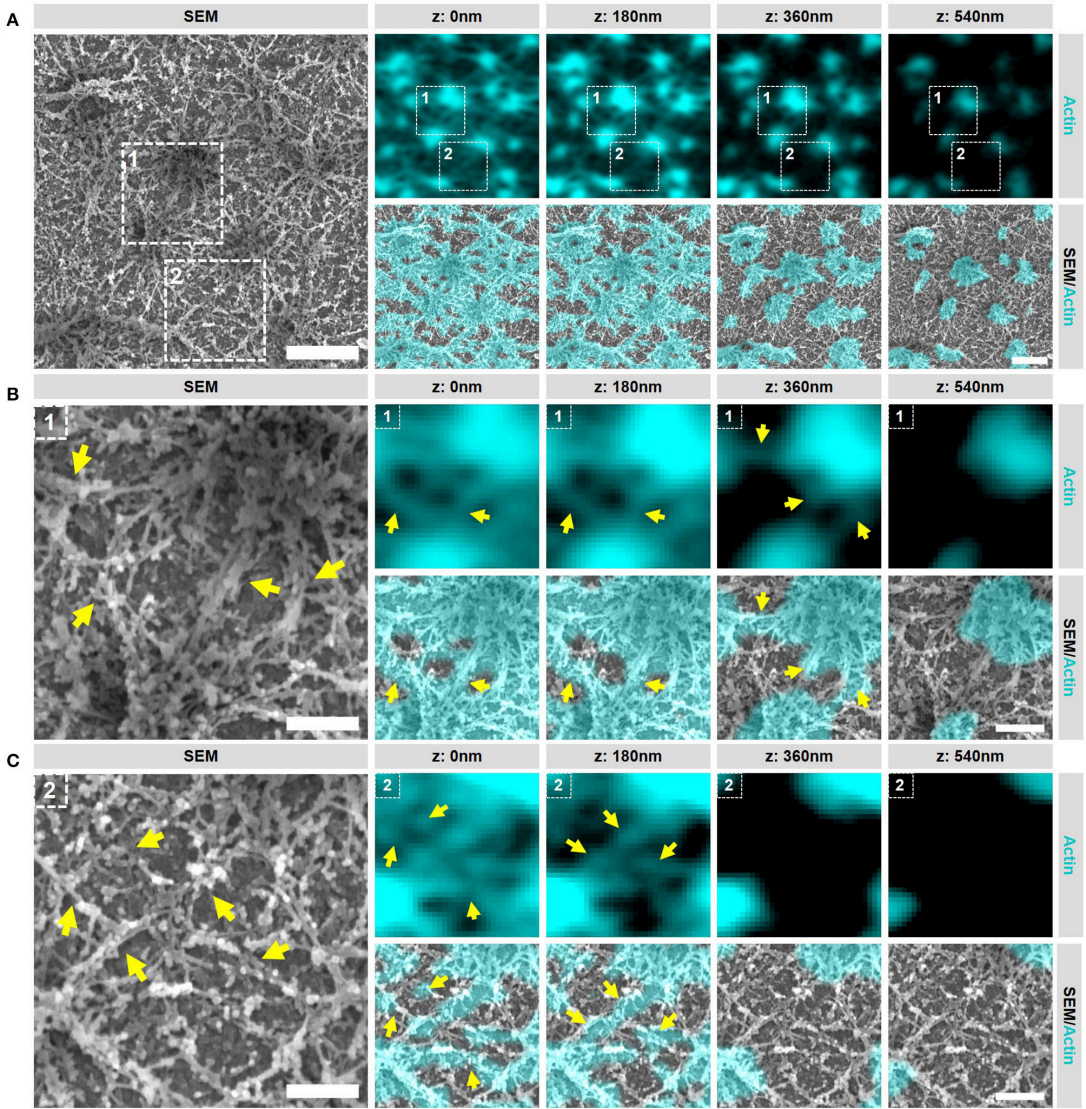
Podosome clusters contain different types of actin filaments. **(A)** DCs were seeded on glass coverslips and after VPM preparation, cells were fixed and stained for actin (cyan). After CPD, DCs were imaged by SEM (gray). Shown is a representative area within a cluster showing the podosome cores and the dense actin network. Scale bar = 1 μm. **(B)** Zoom area number 1 from panel **(A)**. Shown is a representative area that primarily contains actin filaments that are directly associated to podosome cores (indicated by the arrows). Scale bar = 400 nm. **(C)** Zoom area number 2 from panel **(A)**. Shown is a representative area that primarily contains actin filaments that are not directly associated to podosome cores (indicated by the arrows). Scale bar = 400 nm.

Having established our SR-CLEM imaging pipeline, we next aimed to study several podosome characteristics that have proved to be challenging by LM alone. As such, we first investigated the ultrastructural organization of actin in podosome clusters of human DCs. Interestingly, when observed by SEM, the cluster clearly stands out from the rest of the VPM with respect to the density of filamentous structures (Supplementary Figure 3). In fact, the entire cluster appears to be characterized by a very thick layer of filaments and correlating the SEM with the LM indicated that these filaments, as expected, are composed of actin (Figure 3A). Interestingly, at least two types of actin filaments can be identified in the podosome cluster based on SR-CLEM. Firstly, and as anticipated, the cluster contains actin filaments that radiate from the podosome core (Figure 3B). These filaments are associated with the top of the core and run down to the plasma membrane where, in many cases, they appear to associate with filaments that originate from neighboring cores. Secondly, and much less anticipated, there are many filaments that do not appear to be associated with podosome cores (Figure 3C). These filaments are restricted to the ventral side of the plasma membrane and appear to assemble a thick layer of cortical actin that is present at the bottom of the entire podosome cluster.

These results indicate that multiple types and layers of actin filaments are present in the podosome cluster and reveal that the actin network around podosomes in DCs is much more complex than previously anticipated. They further indicate that the SEM ultrastructural information provides a unique insight into the actin organization of podosome clusters in DCs which can be used to further study the localization of podosome associated proteins.

### Vinculin and zyxin associate with different structures in podosomes

**Figure 4:**
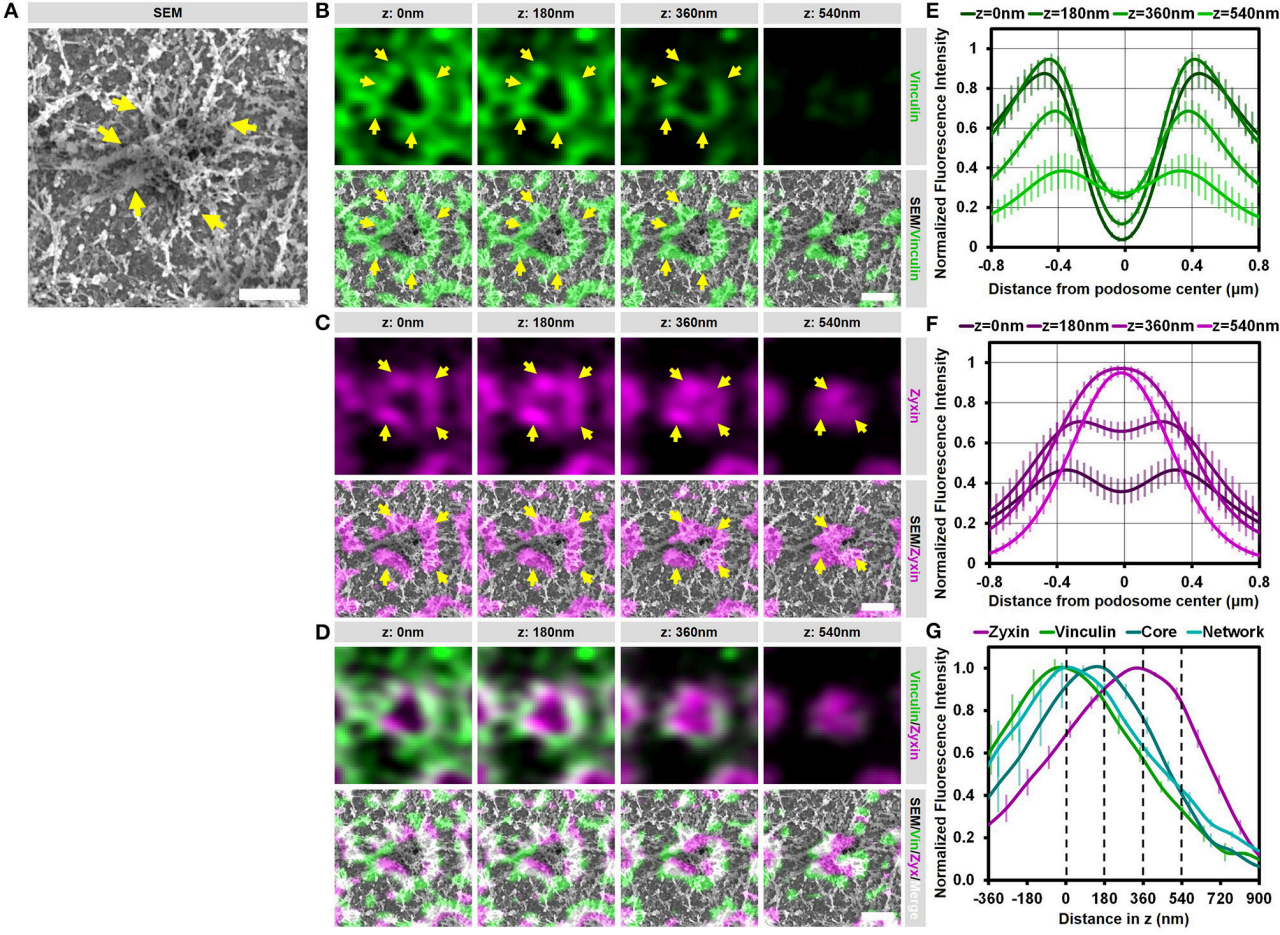
Vinculin and zyxin associate with different structures in podosomes. **(A–D)** DCs were seeded on glass coverslips and after VPM preparation, cells were fixed and stained for vinculin (green, **B**), zyxin (magenta, **C**) and actin (cyan, Supplementary Figure 4). Overlay between vinculin and zyxin is shown in white **(D)**. After CPD, DCs were imaged by SEM (gray, **A**). Shown is a representative podosome with radiating filaments that are clearly observed in the SEM image. Arrows indicate areas enriched for vinculin or zyxin, which overlap with the radiating actin filaments (also indicated by arrows). Scale bar = 500 nm. **(E–F)** Radial fluorescence profile analysis of vinculin **(E)** and zyxin **(F)** at each of the four z-sections depicted in **(B–D)**. Shown is the average ± SEM (*n* = 312 podosomes, 5 cells). **(G)** Quantification of the localization in z of zyxin, vinculin, core actin and network actin in podosomes. The z-sections shown in **(B–D)** are represented by the dashed lines in the graph. Shown is the average ± SEM (*n* = 312 podosomes, 5 cells).

We have shown before that vinculin and zyxin have a different organization in podosome rings, with the vinculin ring being slightly larger compared to the zyxin ring (15). Furthermore, we have shown that vinculin recruitment is dependent on the integrity of the radiating actin filaments while the recruitment of zyxin is not (12). Considering that both these proteins are classified as ring proteins, these differences in the organization and regulation of vinculin and zyxin prompted us to study the detailed organization of these adaptor proteins by our SR-CLEM procedure. Supplementary Figure 4 and Figure 4 show a representative individual podosome with long radiating actin filaments that is located within a large cluster. By analyzing the localization of vinculin, we found that it is very much enriched around the core and clearly present in the ring region. By correlating the vinculin fluorescence with the SEM actin ultrastructural information, we observed that vinculin is especially enriched at sites where the radiating filaments seem to connect the ventral plasma membrane, resulting in a non-continuous ring around the core (Figure 4B, arrows). Unexpectedly, vinculin is also localized to the smaller ventral actin filaments that do not appear to associate with a podosome core (Figure 4B, lower 2 planes). Overall, the 3D Airyscan images clearly indicate that the majority of vinculin is localized very proximal to the plasma membrane. For zyxin, we observed a surprisingly different organization. Firstly, zyxin is almost completely absent from the small actin filaments that do not associate with podosome cores (Figure 4C, lower 2 planes). Secondly, although zyxin, like vinculin, is enriched at sites where the radiating filaments are located, zyxin localizes more closely to the podosome cores and is especially enriched at the top of podosomes (Figure 4C, arrows). Thirdly, zyxin localizes more distal from the plasma membrane compared to vinculin (Figures 4C,D).

To further substantiate our visual inspection, we quantified the localization of vinculin, zyxin, and actin for hundreds of podosomes in multiple cells in both the lateral and the axial direction. For this, fluorescence intensity profiles of vinculin, zyxin and actin were generated both as a function of the distance from the podosome center (Figures 4E,F and Supplementary Figure 4) as well as the distance in z (Figure 4G). First, the intensity profiles as a function of the distance from the podosome center clearly indicated that vinculin is localized more distant from the podosome center compared to zyxin (Figures 4E,F). Importantly, while vinculin localization remains ring-shaped at all z-sections analyzed, zyxin is clearly not at higher planes, strongly suggesting that zyxin is not only associated to the radiating actin filaments but also to the top of the podosome core. Second, the intensity profiles as a function of distance in z clearly indicated that vinculin is localized more close to the membrane compared to zyxin (Figure 4G). While vinculin intensity completely overlaps with the actin network intensity, zyxin intensity peaks at much higher z-sections, most likely corresponding to the top of podosome cores. Importantly, we performed the same quantitative analysis in intact cells and observed a very similar organization for vinculin (Supplementary Figure 5), zyxin and actin suggesting that our results on the VPMs are not a result from an artifact from the complex sample preparation.

Together, these correlative data and quantitative analyses reveal that vinculin and zyxin have a very different organization and strongly suggest that vinculin is specifically recruited to sites where actin filaments connect to the membrane while zyxin appears to decorate the podosome core from the middle to the top.

### Closely associated podosomes are connected by actin filaments positive for zyxin

**Figure 5:**
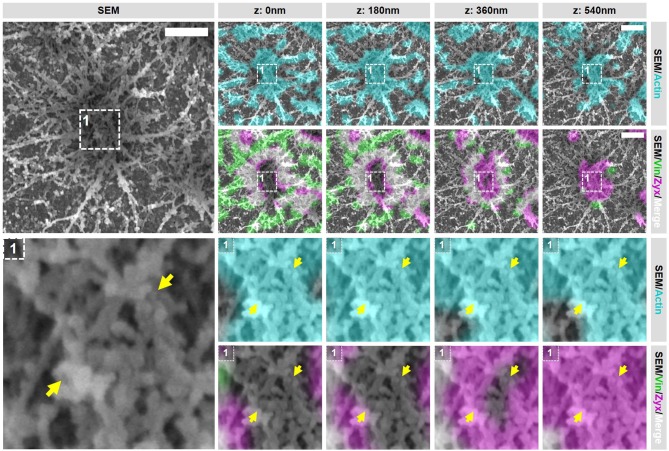
Closely associated podosomes are connected by actin filaments positive for zyxin. DCs were seeded on glass coverslips and after VPM preparation, cells were fixed and stained for vinculin (green), zyxin (magenta) and actin (cyan). Overlay between vinculin and zyxin is shown in white. After CPD, DCs were imaged by SEM (gray). Shown is a representative image of two closely associated podosomes that share one vinculin ring (Supplementary Figure 6 shows an additional example and corresponding individual LM channels are shown in Supplementary Figure 7). Arrows indicate a thick bundle of linear actin filaments that stretch from one core to the other. Scale bar = 500 nm.

Podosomes undergo continuous fission and fusion events resulting in closely associated neighboring podosomes (19, 20). These events are mechanistically very poorly understood and we therefore decided to study them in greater detail with our SR-CLEM procedure. Importantly, for this study, podosomes were defined as closely associated when two separate actin cores were surrounded by one continuous vinculin ring. With these criteria, we selected two representative events of closely associated podosomes (Figure 5 and Supplementary Figures 6, 7). Firstly, we observed that, similar to individual podosomes described above, vinculin and zyxin are very differently organized in these closely associated podosomes. Vinculin is present proximal to the plasma membrane and associates with the actin filaments that radiate from the two podosome cores while zyxin is localized more closely to the two cores (Figure 5, Supplementary Figures 6, 7). Moreover, while at this stage, vinculin is present around the two cores and not in between, resulting in one continuous vinculin ring around the two actin cores, zyxin is clearly associated with and entirely covers the two separate cores (Figure 5, Supplementary Figure 6). Together, these data strengthen the notion that vinculin and zyxin have very different functions at podosomes. While vinculin may specifically stabilize the filaments around podosome cores, zyxin is likely involved in stabilizing filaments that cover the podosome core.

Importantly, when closely analyzing the SEM ultrastructural information of these closely associated podosomes, we noted a structure that has not been described before. In both representative examples, the closely associated podosomes appear to be connected by a thick bundle of linear actin filaments that stretch from one core to the other (Figure 5 and Supplementary Figure 6, arrows). This bundle of actin filaments was almost always observed [41 out of 44 (93%) events analyzed], strongly suggesting that this structure is a general and important feature of podosomes that are closely associated. Interestingly, these filaments are associated with zyxin at the top but not with vinculin suggesting that they do not associate with the plasma membrane and may originate from the core actin.

### Substrate topology induces interpodosomal connections

**Figure 6:**
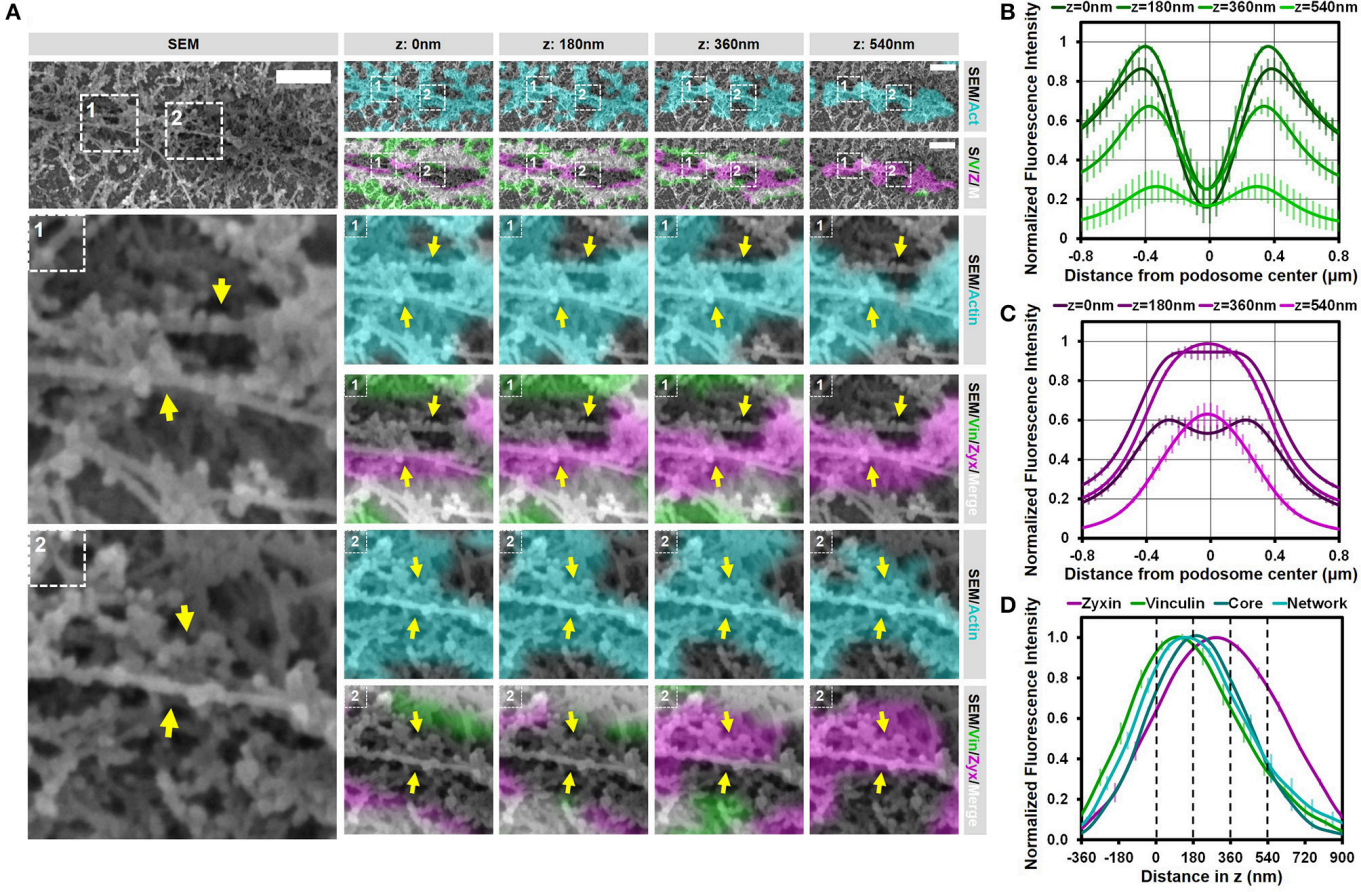
Substrate topology induces interpodosomal connections. **(A,B)** Radial fluorescence profile analysis of vinculin **(A)** and zyxin **(B)** of podosomes on topographical cues for each of the four z-sections depicted in **(D)**. Shown is the average ± SEM (*n* = 140 podosomes, 4 cells). **(C)** Quantification of the localization in z of zyxin, vinculin, core actin and network actin in podosomes on topographical cues. The z-sections shown in **(D)** are represented by the dashed lines in the graph. Shown is the average ± SEM (*n* = 140 podosomes, 4 cells). **(D)** DCs were seeded on manually scratched glass coverslips and after VPM preparation, cells were fixed and stained for vinculin (green), zyxin (magenta) and actin (cyan). Overlay between vinculin and zyxin is shown in white. After CPD, DCs were imaged by SEM (gray). Shown is a representative area that contain multiple (at least 3) closely associated podosomes aligned on a topological feature that share one podosome ring (Supplementary Figure 9 shows an additional example and corresponding individual LM channels are shown in Supplementary Figure 10). Arrows indicate the thick bundle of linear actin filaments that stretch from one core to the other. Scale bar = 500 nm.

We previously demonstrated that substrate topographical cues induce the close association of podosomes (21). To study if this induced association of podosomes by substrate topology is similar to the closely associated podosomes on flat surfaces, we seeded DCs on coverslips with manually made scratches and investigated the organization of podosomes on top of these scratches with SR-CLEM. As shown before, we first noticed that the surface topography induced the alignment of podosomes and their close association (Supplementary Figure 8). To analyze the localization of vinculin and zyxin as well as the SEM ultrastructural information, we selected two representative arrays of multiple closely associated podosomes, where the different actin cores could still be recognized (Figure 6A and Supplementary Figures 9, 10). Interestingly, similar to the closely associated podosomes on a flat surface, vinculin was specifically present at the actin filaments around the cores but not in between, resulting in one large vinculin ring encircling sometimes as many as 5 actin cores (Supplementary Figure 8). Also for zyxin, we noted a similar localization when compared to the podosomes on a flat surface, with zyxin being closely associated with the side and top of each individual podosome core.

To substantiate these findings for podosomes on topographical cues, we generated fluorescence intensity profiles as a function of the distance from the podosome center (Figure 6B,C and Supplementary Figure 11) and the distance in z (Figure 6D). The intensity profiles as a function of the distance from the podosome center were very similar to those on flat surfaces, demonstrating that, also on topographical cues, zyxin is localized more closely to the podosome core as compared to vinculin. Interestingly, while the intensity profiles as a function of distance in z clearly indicated that the proteins were separated in the axial direction similar to the proteins on flat surfaces, the differences were much smaller on topographical cues, something which may be caused by the core protruding in the manually made scratch. Overall, our results indicate that the topographical cues induces only small changes in the localization of the adaptor proteins despite the variety in actin structures induced by the topographical cues.

We next investigated whether the interpodosomal connections observed on a flat surface were also present between podosome cores on topographical cues. Interestingly, we indeed clearly observed linear actin filaments that seem to connect two neighboring cores in our representative examples (Figure 6A and Supplementary Figure 9, arrows). Quantitative analysis showed that in 41 out of 41 (100%) events, these linear actin filaments were present, suggesting that, also on topographical cues, these filaments play an important role in regulating the close association of two neighboring podosomes. Moreover, these actin filaments were positive for zyxin but not for vinculin, suggesting that the nature of these filaments on the topographical cues is very similar compared to a flat surface. These filaments may therefore be a general feature of podosomes that are closely associated, and possibly undergo fission or fusion.

### Actin polymerization essential for the integrity of vinculin-associated filaments but not for zyxin-associated interpodosomal connections

So far, our results strongly suggest that vinculin and zyxin primarily associate to different actin structures within podosome clusters. To investigate whether these different actin structures have a different dynamic regulation, we performed SR-CLEM on cells treated with CytoD, an inhibitor of actin polymerization. We have shown before that inhibition of actin polymerization by CytoD causes the immediate disappearance of vinculin and the actin filaments from podosome clusters while zyxin and the podosome cores remain present for prolonged times (12). We therefore first examined the localization of vinculin, zyxin and actin in podosomes by SR microscopy after the inhibition of actin polymerization and confirmed these published observations (Supplementary Figures 12, 13). It should be noted though that, while zyxin localization is still mostly intact, the ring-shaped localization is less pronounced and the distance between the remaining network and zyxin is decreased after treatment with CytoD (Supplementary Figure 13), indicating that minor changes occur in the organization of the podosome core and associated zyxin after inhibition of actin polymerization.

**Figure 7:**
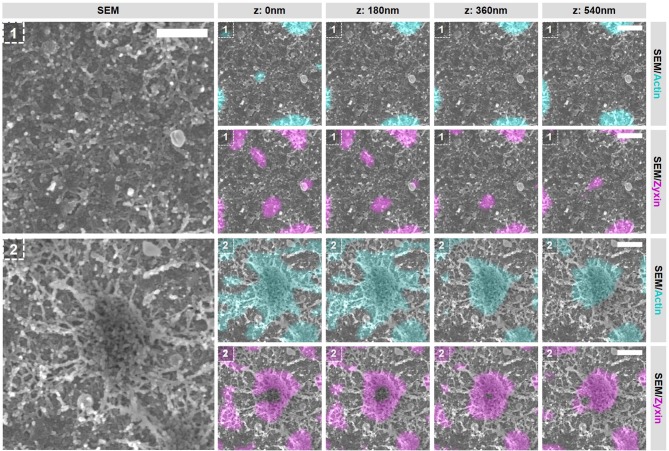
Actin polymerization essential for the integrity of vinculin-associated filaments. DCs were seeded on manually scratched glass coverslips, treated with CytoD for 10 min and after VPM preparation, cells were fixed and stained for actin (cyan), zyxin (magenta) and vinculin (not shown in this Figure). After CPD, DCs were imaged by SEM (gray). Shown are two areas that represent (1) an area in between podosomes and (2) an individual podosome in a podosome cluster (Supplementary Figure 14 shows the entire cell from which the areas were selected and Supplementary Figure 16 shows the individual LM images). Scale bar = 400 nm.

To evaluate the integrity of the actin structures within podosome clusters after the inhibition of actin polymerization, we first selected 2 areas in the podosome cluster representing (1) an area in between podosome cores and (2) an individual podosome within the cluster (Figure 7 and Supplementary Figures 14, 16). Firstly, we observed large changes in the area in between podosome cores (Figure 7, region 1). Whereas comparable areas in non-treated cells are completely covered with actin filaments that do not appear to be associated with podosomes (Figure 3C), these have all disappeared after inhibition of actin polymerization. Secondly, we noted that also the large filaments that connect to podosome cores have mostly disappeared (Figure 7, region 2). We did observe some actin filaments that remained associated to the core but these are mostly present at the bottom and no longer at the top of podosome cores. Together, these results indicate that a majority of the actin filaments that appeared to be vinculin-associated by our SR-CLEM observations have disappeared after inhibition of actin polymerization and may therefore reflect the most dynamic pool of actin within the podosome cluster. Interestingly, by SEM, we observed that the podosome core itself appeared to be mostly intact (Figure 7, region 2), correlating with the prolonged presence of zyxin and suggesting that the core actin is less dynamic.

**Figure 8:**
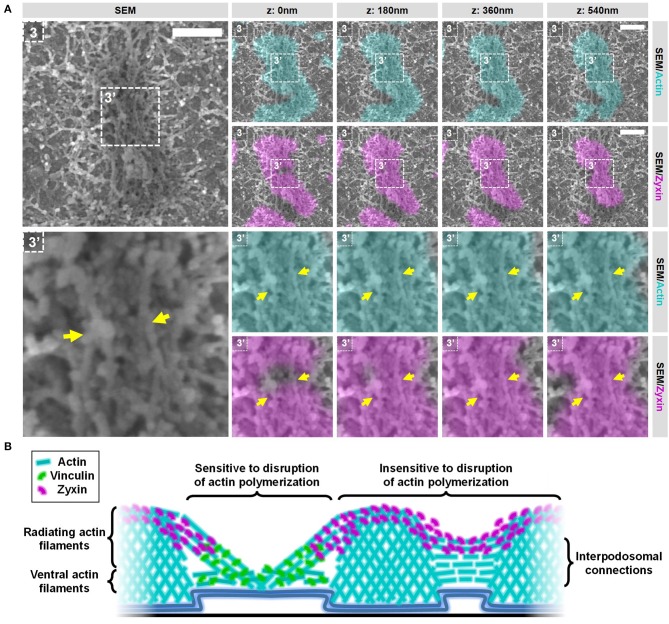
Actin polymerization not essential for the integrity of the zyxin-associated interpodosomal connections. **(A)** DCs were seeded on manually scratched glass coverslips, treated with CytoD for 10 min and after VPM preparation, cells were fixed and stained for actin (cyan), zyxin (magenta) and vinculin (not shown in this Figure). After CPD, DCs were imaged by SEM (gray). Shown is a representative event of two closely associated podosomes on a flat surface. (Supplementary Figure 14 shows the entire cell from which this event was selected and Supplementary Figure 16 shows the individual LM images). Arrows indicate the thick bundle of linear actin filaments that stretch from one core to the other. Scale bar = 400 nm. **(B)** Schematic model of the different actin structures and the localization of vinculin and zyxin as observed by SR-CLEM.

Next, we selected 2 areas representing closely associated podosomes on a flat surface and a topographical cue (Figure 8 and Supplementary Figures 15, 16) to study the integrity of the interpodosomal connections after inhibition of actin polymerization. Strikingly, and in sharp contrast to the vinculin-associated filaments, we still very frequently observed interpodosomal connections between two closely associated podosomes, both on flat surfaces (Figure 8, arrows) and on topographical cues (Supplementary Figure 15, arrows). Although our initial criteria could not be used in identifying closely associated podosomes on flat surfaces since vinculin was completely absent, we still observed 26 interpodosomal connections in 4 cells on flat surfaces and in 20 out of 22 neighboring podosomes (91%) on topographical cues, which was very much comparable to our observations in untreated cells. Interestingly, these interpodosomal connections did not only appear to be completely intact based on inspection of the SEM image, they were also still associated with zyxin (Figure 8 and Supplementary Figure 15). Together, these data indicate that the interpodosomal connections are resistant to the inhibition of actin polymerization and may represent a less dynamic pool of actin that is different in nature compared to the vinculin-associated ventral or radiating filaments.

## Discussion

We here developed and optimized a correlative multicolor Airyscan super-resolution and SEM imaging pipeline to study the organization of the ventral plasma membrane. With our procedure, we achieve a near perfect overlay of at least three fluorescent channels with a SEM image across entire cells and reveal novel insights into the ultrastructural organization of podosomes in DCs. We show that the actin network at podosome clusters is more dense and complex than previously anticipated. Further, we find that zyxin, classically known as a podosome ring protein, appears to be more associated with the core. Also, we show that closely associated podosomes on flat surfaces as well as topographical cues are connected by thick actin filaments that are associated with zyxin, but not with vinculin. Finally, we show that actin filaments present in the podosome cluster have a differential sensitivity to inhibition of actin polymerization suggesting that they are dynamically distinct pools of actin.

We show that podosome clusters in DCs contain many different types and layers of actin filaments (Figure 8B), which is clearly different from the rest of the cell where the actin network is much thinner. While some of the identified filaments in the podosome cluster clearly associate with the side of podosome cores, others do not, something which has not been described before. Filaments that do not associate with the side of the core are much smaller and located closer to the plasma membrane. Based on our data, these filaments could either be not associated to podosome cores at all or only at the bottom of podosomes. The complex organization of dense actin filaments around podosomes was already shown for the sealing zone in osteoclasts (9). The sealing zone, however, is a specialized bone resorbing organelle in osteoclasts, and it was unclear to what extent its actin organization could be compared to podosome clusters in other cell types. We showed before that the enrichment of αMβ2 integrins and talin specifically delineates the podosome cluster in DCs (8). Together with the specialized actin organization presented here, these results clearly demonstrate that the podosome cluster in DCs is a region at the ventral plasma membrane that should be considered as a separate regulatory platform. For future experiments, it would be interesting to focus on unraveling the exact nature of these different filaments and how they contribute to the different podosome functions in DCs such as protrusion and mechanosensing. We expect that the large core-associated actin filaments stabilize podosomes cores and contribute to protrusion while the small actin filaments close the plasma membrane may have a function in vesicle transport within the podosome cluster or the positioning of the podosome cores.

Vinculin and zyxin are two adaptor proteins that are classically associated with the podosome ring (5). Using our SR-CLEM procedure, we here show that zyxin is in fact for the most part associated with the podosome core rather than the ring (Figure 8B). This novel finding on the differential organization of vinculin and zyxin further extends our previous findings that zyxin is located more close to the core than vinculin (15) and that zyxin localization in podosomes is not dependent on the integrity of the actin filaments that are located in the ring region (12). Furthermore, our result that zyxin is located in a higher vertical plane than vinculin matches the nanoscale organization of these proteins in focal adhesions (16), indicating structural similarities between the radiating filaments and focal adhesions. Interestingly, the localization of zyxin in podosomes that we reveal here is very reminiscent of podosome cap proteins such as supervillin (22) and LSP-1 (23). We therefore propose that zyxin is present in the podosome cap mainly at site where the filaments radiate from the core. In the cap, zyxin could be involved in the repair of actin filaments that are under high stress due to the protrusive forces of podosomes, as has been shown for strained stress fibers (14). Next step would be to unravel the mutual localization of other podosome components with our SR-CLEM procedure, which may also have been misclassified.

Podosomes continuously split and fuse but the function and mechanism for these events are very poorly understood. One study shows that podosome fission is a means of new podosome assembly at the leading edge of macrophages (19). Furthermore, it was shown that fission and fusion are regulated by microtubules and are therefore thought to be active processes that are tightly regulated (19, 20). Here, we identify a novel actin-based structure that connects two closely associated podosome cores, which are likely in the process of fusion or fission since they share a vinculin ring. This structure has not been observed before, probably due to the density of actin features in LM images. This newly identified structure also connects closely associated podosomes that align along the edges of topographical cues suggesting that this structure may have a general function in interpodosomal communication and podosome mechanosensing. Interestingly, the integrity of this structure appeared to be insensitive for inhibition of actin polymerization suggesting that it represents a slow dynamic pool of actin that is clearly different from actin in the radiating actin filaments, which rapidly lose their integrity after actin polymerization is blocked. Lastly, the fact that this structure is positive for zyxin suggests that this structure may be under tension in steady state conditions, possibly created by pushing and pulling from the two podosome cores. It would now be particularly interesting to classify these closely associated podosomes by performing VPM preparation immediately after single cell live imaging, to reveal whether this novel structure is particularly important for fusion, fission or both.

In conclusion, we here demonstrate that our novel SR-CLEM imaging pipeline is a valuable tool for revealing the mutual localization of different proteins in complex multimolecular structures. Using our workflow, we were able to reveal novel ultrastructural details of podosomes in DCs which would have been extremely challenging by LM alone. To provide an even more detailed view on the organization of multiple proteins with respect to the actin ultrastructure, effort should now be put in adapting our imaging pipeline for correlating multicolor 3D-STORM with SEM, theoretically feasible with our procedure since the LM is performed under wet conditions. Lastly, we envisage that our workflow can further be used to study the organization of other multimolecular structures at the ventral plasma membrane such as clathrin coated pits, the cortical actin network, focal adhesions and the immune synapse.

## Ethics statement

All experiments involving human material were carried out after obtaining written informed consent from all subjects as per the Declaration of Helsinki. The study was approved by the Institutional Review Board of the Radboud University Nijmegen Medical Center, Commissie Mensgebonden Onderzoek.

## Author contributions

BJ optimized the SR-CLEM imaging pipeline, performed all experiments and acquired the images. MW, JF, AC, and KvdD provided input for optimizing the SR-CLEM imaging pipeline. BJ and KvdD optimized image alignment and the LM-SEM merge procedure. BJ, AC, and KvdD designed the study and interpreted the data. AC and KvdD supervised the study. KvdD prepared the figures and wrote the manuscript with input from all authors.

### Conflict of interest statement

The authors declare that the research was conducted in the absence of any commercial or financial relationships that could be construed as a potential conflict of interest.
