# Supplementary material for: Super-Resolution Correlative Light and Electron Microscopy (SR-CLEM) Reveals Novel Ultrastructural Insights Into Dendritic Cell Podosomes
Source: Front Immunol. 2018 Aug 22;9:1908. doi: 10.3389/fimmu.2018.01908 (PMC6113363; doi:10.3389/fimmu.2018.01908)
Supplement: Supplementary file 1 [file Presentation_1.PDF]

Supplementary Material

Super-resolution correlative light and electron microscopy (SR-CLEM) reveals novel ultrastructural insights into dendritic cell podosomes

Ben Joosten, Marieke Willemse, Jack Fransen, Alessandra Cambi and Koen van den Dries*

*Corresponding author: Dr. Koen van den Dries, koen.vandendries@radboudumc.nl

Joosten et al. Supplementary Figure 1

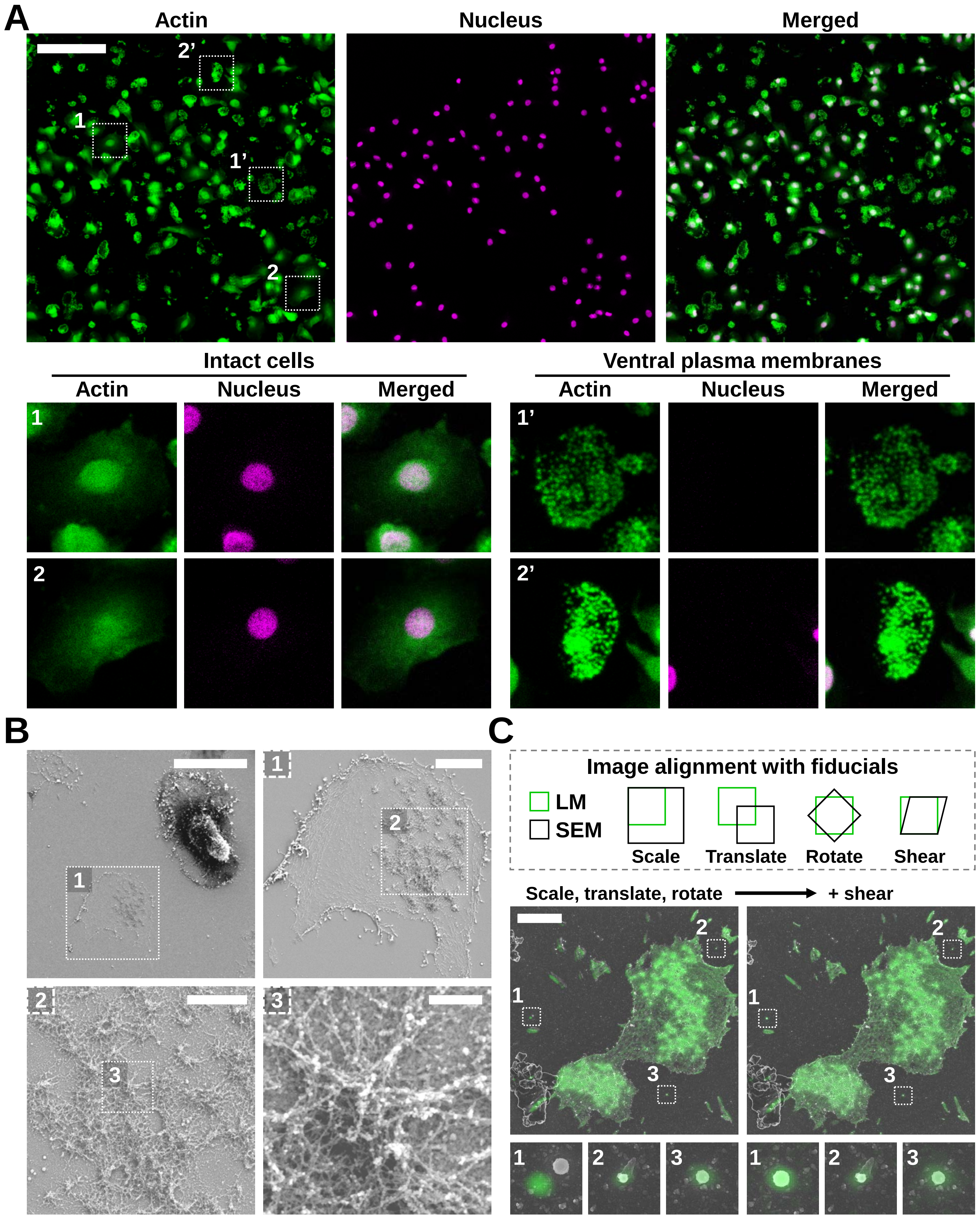

Supplementary Figure 1. Efficiency of VPM preparation and optimization of image alignment

A) DCs were seeded on glass coverslips and sonicated to prepare VPMs. Next, cells were immediately fixed, and labeled for actin (green) and the nucleus (magenta). Shown is a sample overview (top) and zoom in images depicting two intact cells and two ventral plasma membranes. Scale bar = 100 µm. **B)** After CPD, DCs were imaged by SEM (gray). Shown in the top left image is a SEM image of an intact cell and a ventral plasma membrane (area 1). Area 2 depicts a large part of the podosome cluster and area 3 depicts a single podosome. Scale bar = 20 µm (top left), 5 µm (area 1), 2.5 µm (area 2) and 0.5 µm (area 3). **C)** Overview of the image alignment procedure with fiducials. Scale bar = 5 µm.

Joosten et al. Supplementary Figure 2

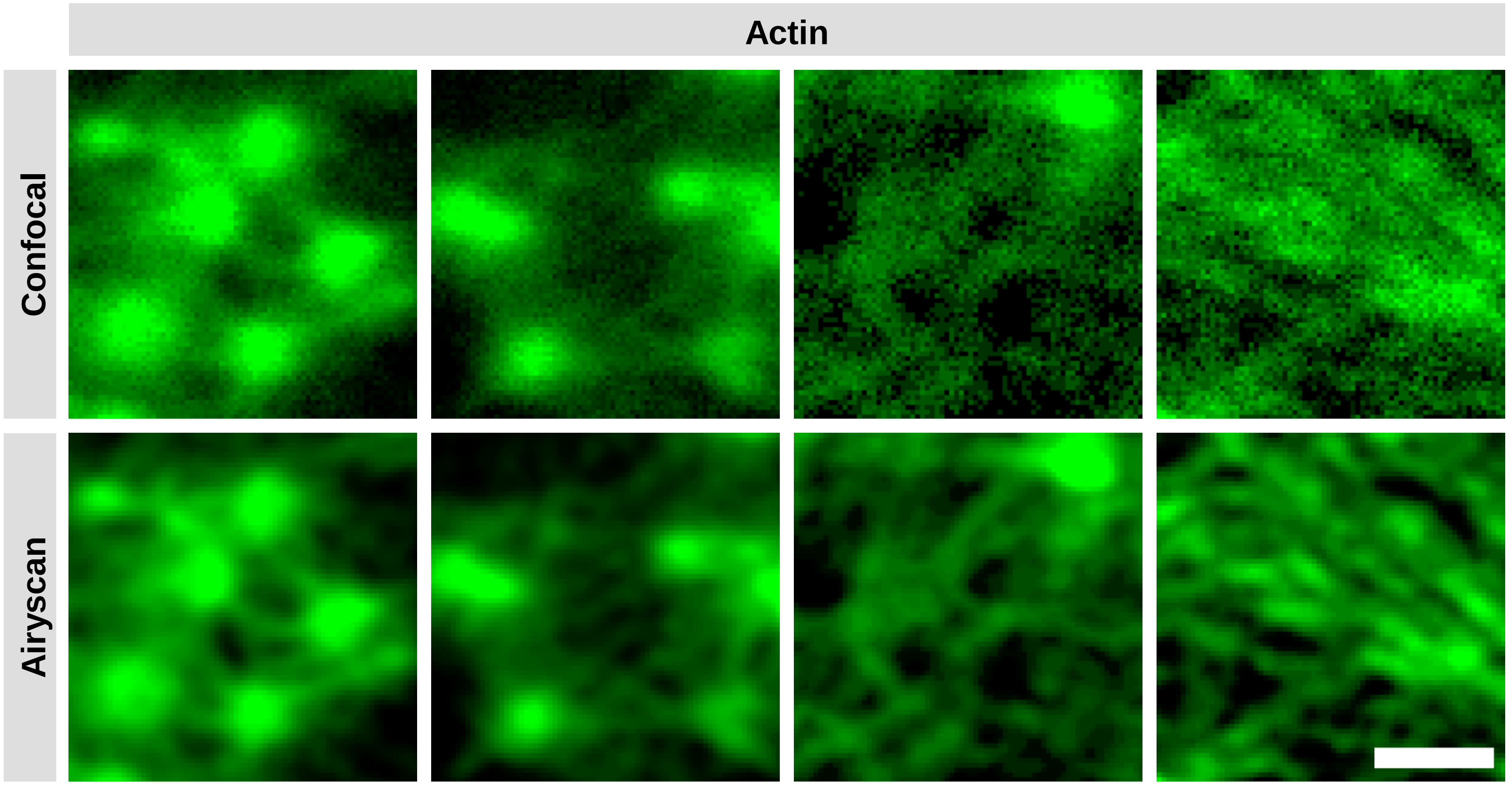

Supplementary Figure 2. Direct comparison of laser scanning confocal and Airyscan microscopy

DCs were seeded on glass coverslips and sonicated to prepare VPMs. Next, cells were immediately fixed and labeled for actin (green). Shown are areas with actin structures imaged by laser scanning confocal (top row) and Airyscan microscopy. Scale bar = 1 μm.

Joosten et al. Supplementary Figure 3

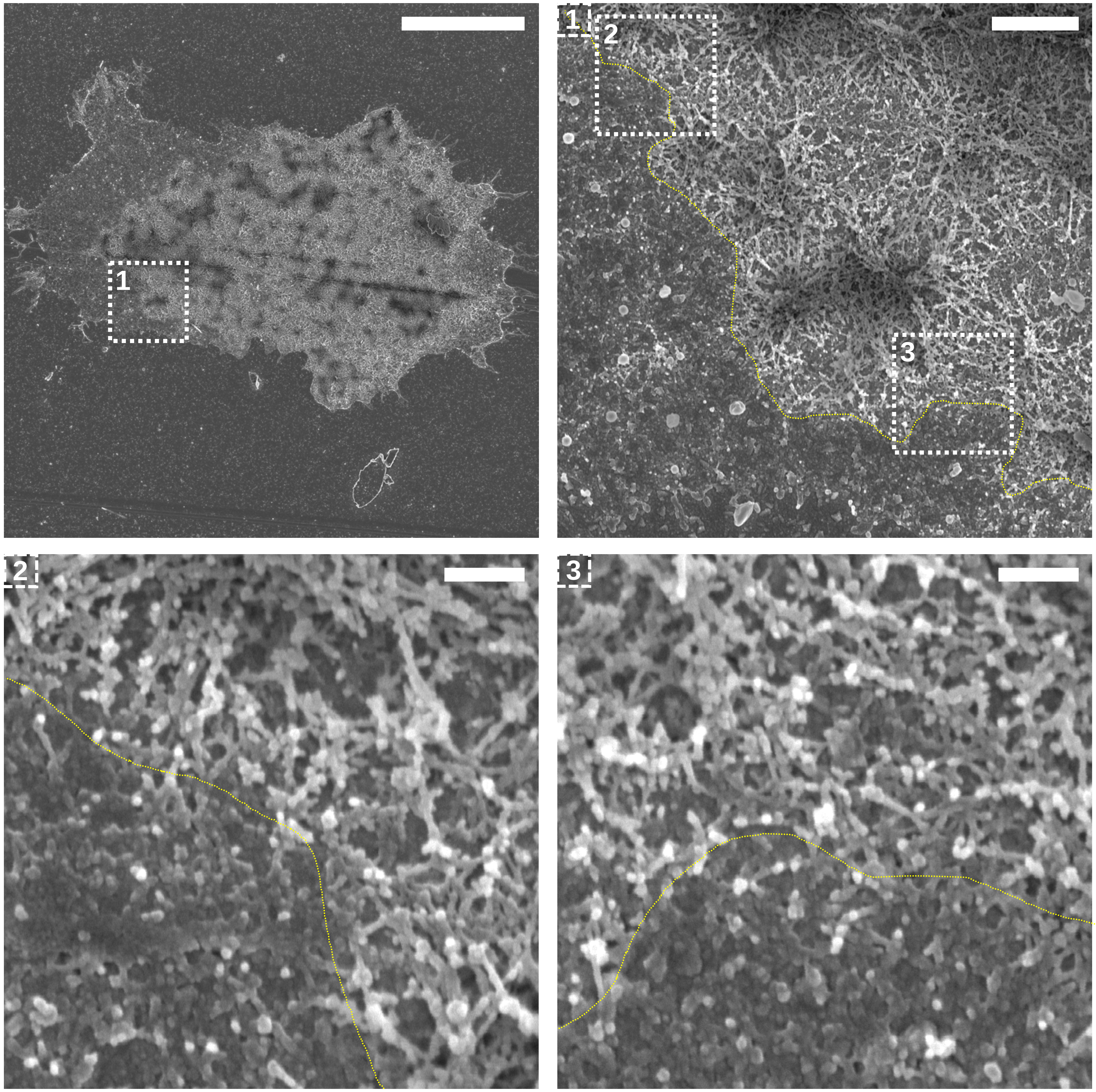

Supplementary Figure 3. Enrichment of actin in podosome clusters

DCs were seeded on glass coverslips, sonicated to prepare VPMs and immediately fixed. After CPD, DCs were imaged by SEM (gray). Shown on the top left is a SEM image of a ventral plasma membrane containing a podosome cluster. Area 1, 2 and 3 depict areas at the edge of the podosome cluster. Podosome cluster edge is indicated by the yellow dotted line. Scale bar = 10 µm (top left), 2 µm (area 1) and 0.5 µm (area 2 and 3).

Joosten et al. Supplementary Figure 4

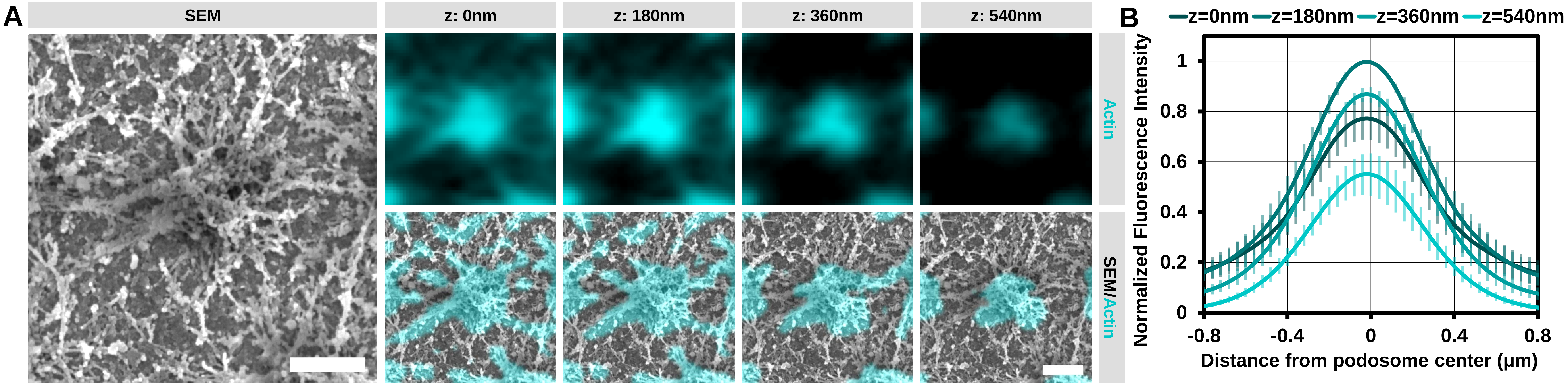

Supplementary Figure 4. Actin organization in individual podosome in cluster

A) Shown is the actin (top row) and SEM/actin merged (bottom row) image corresponding to the podosome shown in main Figure 4. Scale bar = 0.5 µm. **B)** Radial fluorescence profile analysis of actin at each of the four z-sections depicted in **A**. Shown is the average ± SEM (n = 312 podosomes, 5 cells).

Joosten et al. Supplementary Figure 5

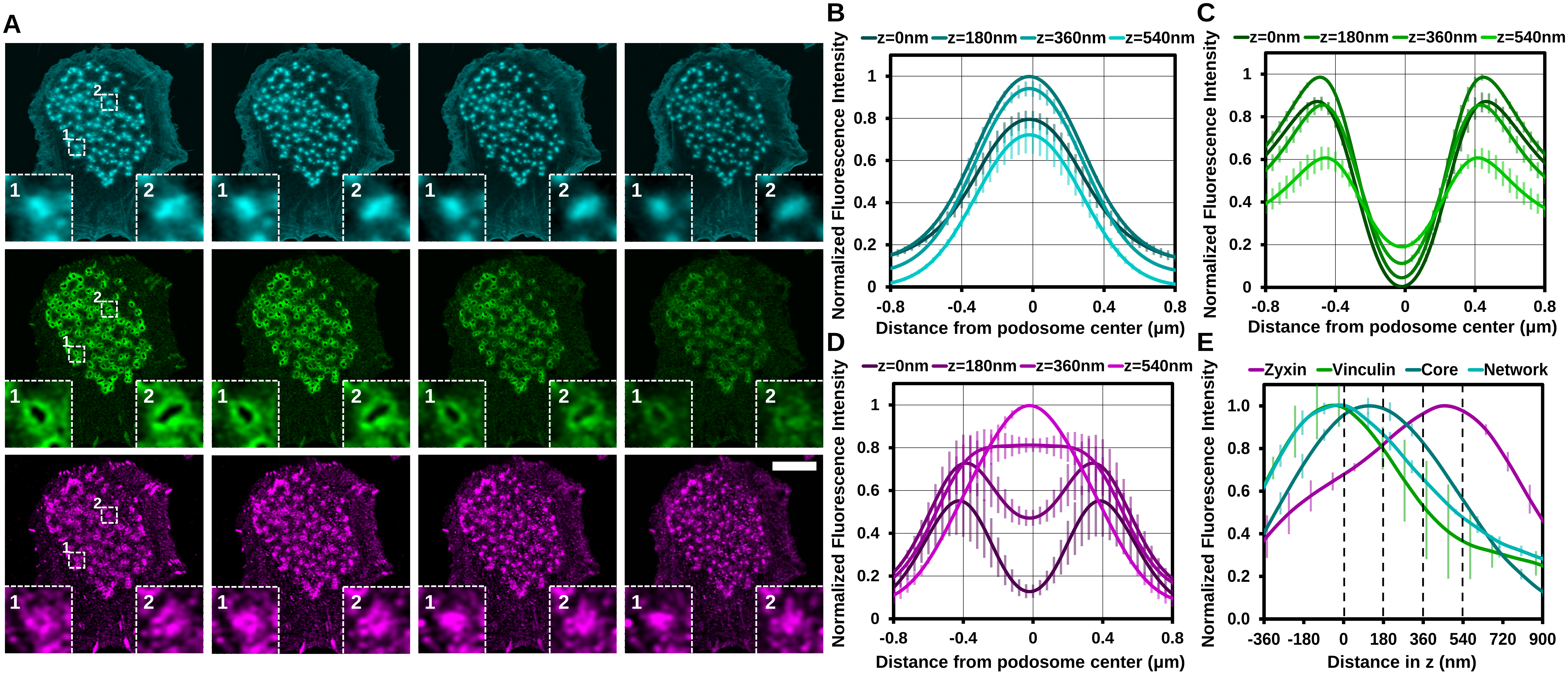

Supplementary Figure 5. Actin, vinculin and zyxin organization in podosome clusters in intact cells

A) DCs were seeded on glass coverslips, fixed and stained for actin (cyan), vinculin (green) and zyxin (magenta). Shown are representative images of all three channels in 3 dimensions. Insets depict two representative podosomes within the cluster. Scale bar = 10 µm. **B-D)** Radial fluorescence profile analysis of actin (**B**), vinculin (**C**) and zyxin (**D**) at each of the four z-sections depicted in **A**. Shown is the average ± SEM (n = 198 podosomes, 2 cells). **E)** Quantification of the localization in z of zyxin, vinculin, core actin and network actin in podosomes in intact cells. The z-sections shown in **A** are represented by the dashed lines in the graph. Shown is the average ± SEM (n = 198 podosomes, 2 cells).

Joosten et al. Supplementary Figure 6

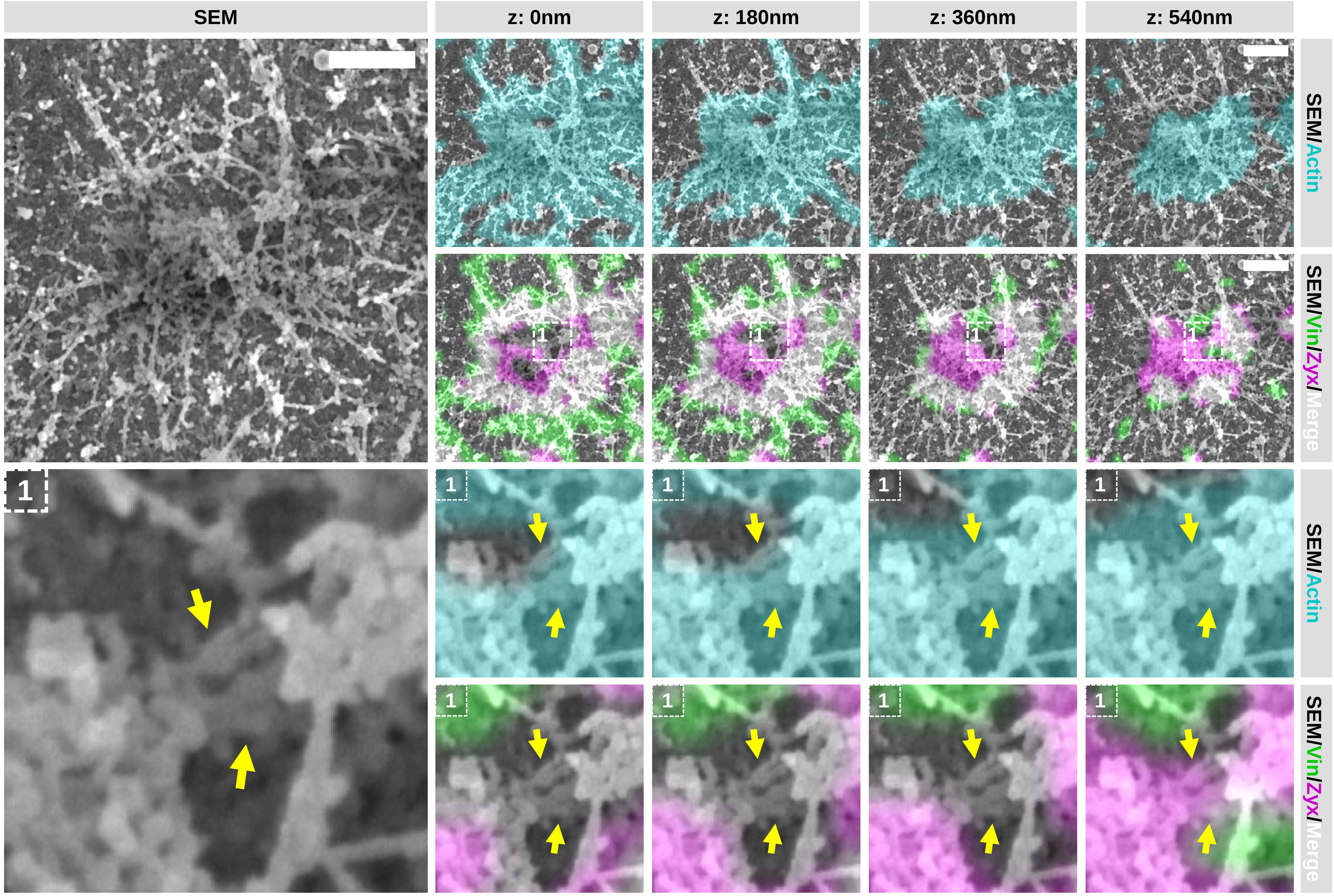

Supplementary Figure 6. Closely associated podosomes are connected by actin filaments positive for zyxin

DCs were seeded on glass coverslips and after VPM preparation, cells were fixed and stained for vinculin (green), zyxin (magenta) and actin (cyan). Overlay between vinculin and zyxin is shown in white. After CPD, DCs were imaged by SEM (gray). Shown is a representative image of two closely associated podosomes that share one vinculin ring (Figure 5 shows an additional example and corresponding individual LM channels are shown in Supplementary Figure 7). Arrows indicate a thick bundle of linear actin filaments that stretch from one core to the other. Scale bar = 500 nm.

Joosten et al. Supplementary Figure 7

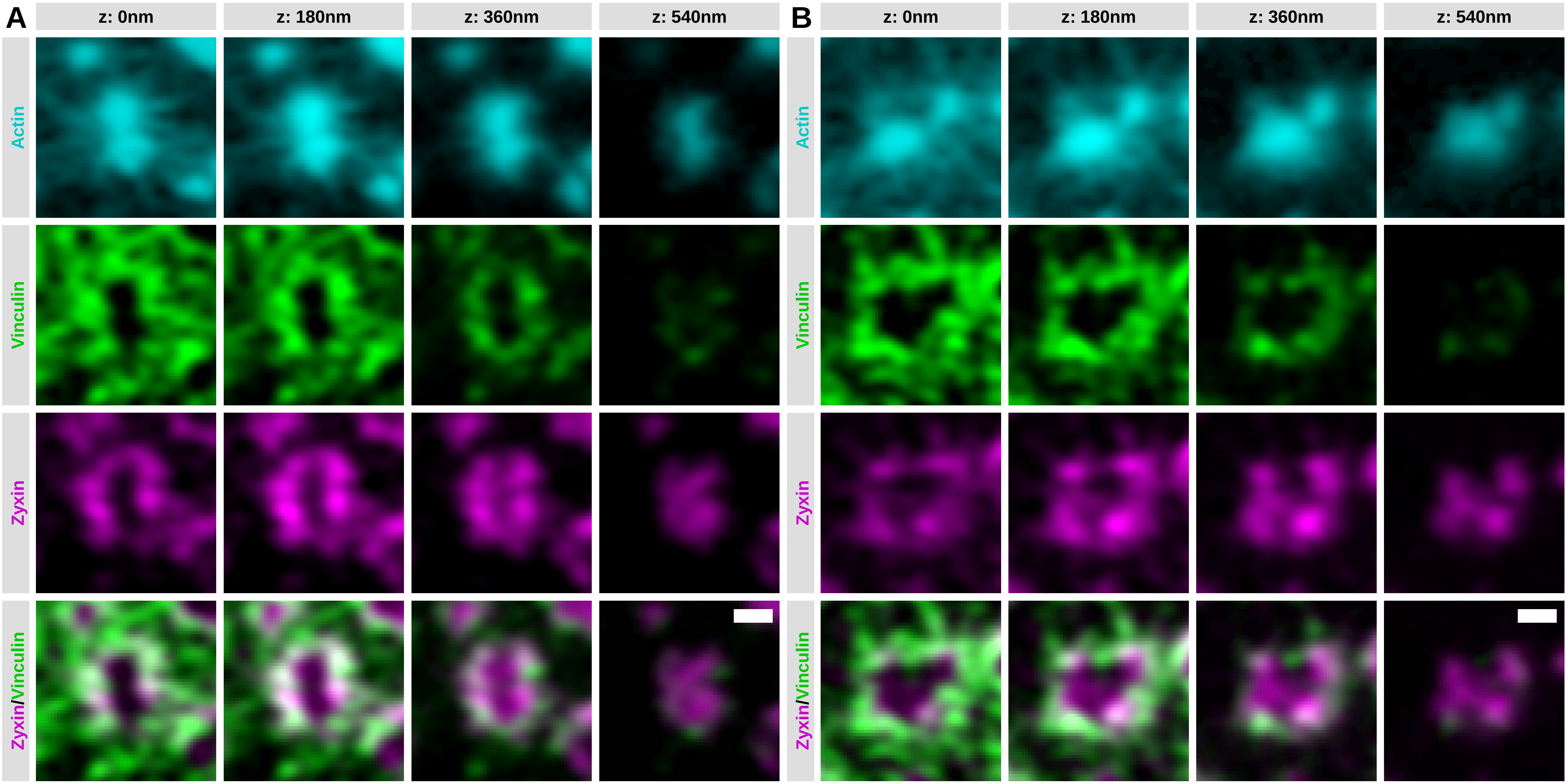

Supplementary Figure 7. Actin, vinculin and zyxin organization in closely associated podosomes on a flat surface
A-B) Shown are the individual LM images for actin (cyan), vinculin (green) and zyxin (magenta) as well as the vinculin/zyxin overlay corresponding to the podosomes shown in main Figure 5 (**A**) and Supplementary Figure 6 (**B**). Scale bar = 0.5 µm.

Joosten et al. Supplementary Figure 8

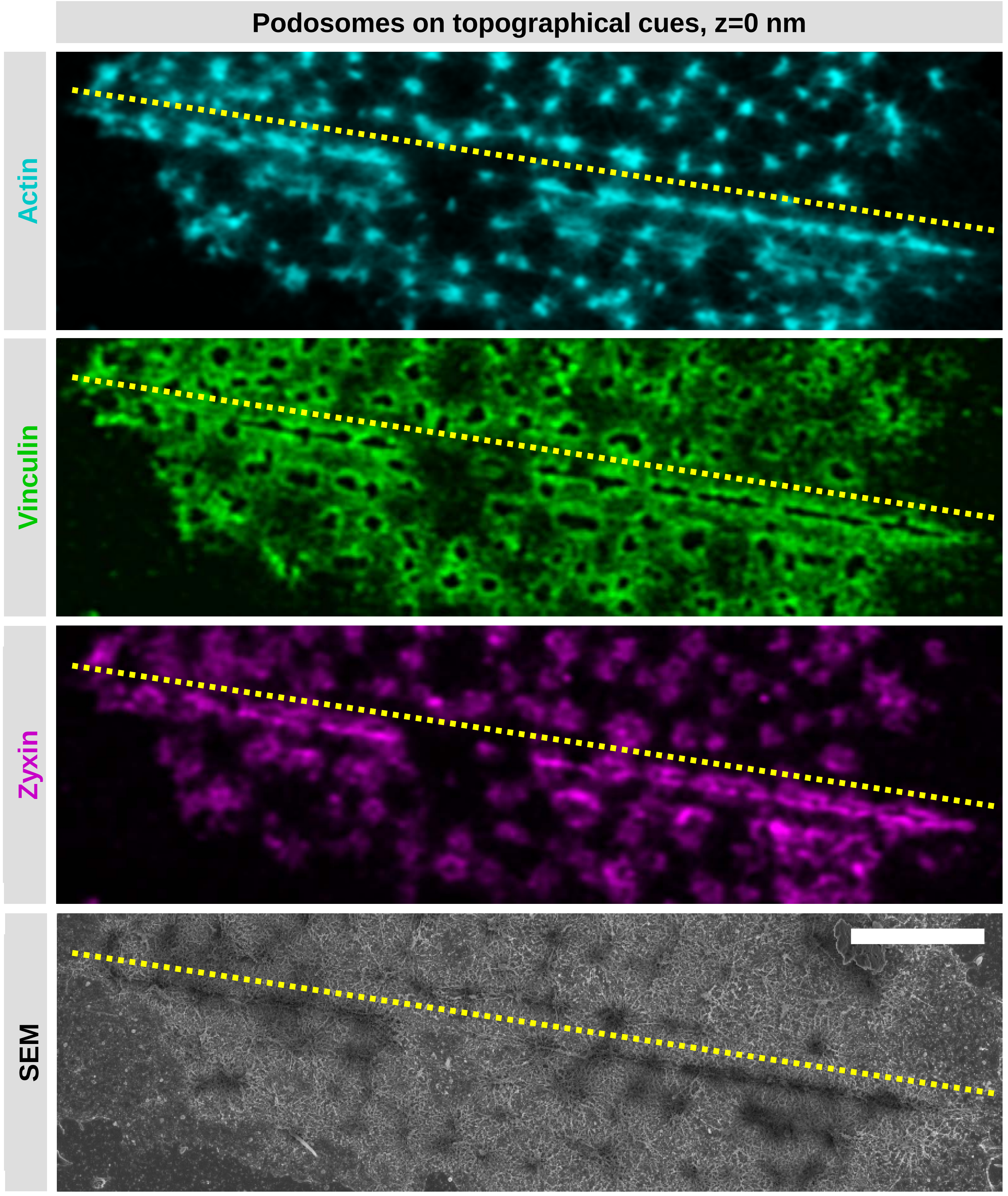

Supplementary Figure 8. Podosomes align on substrate topographical cues

DCs were seeded on manually scratched glass coverslips and after VPM preparation, cells were fixed and stained for actin (cyan), vinculin (green) and zyxin (magenta). After CPD, DCs were imaged by SEM (gray). Shown is a overview of multiple podosomes that align on the topographical cue. The dashed yellow line is drawn right above the topographical cue and indicates its orientation. Scale bar = 5 μm.

Joosten et al. Supplementary Figure 9

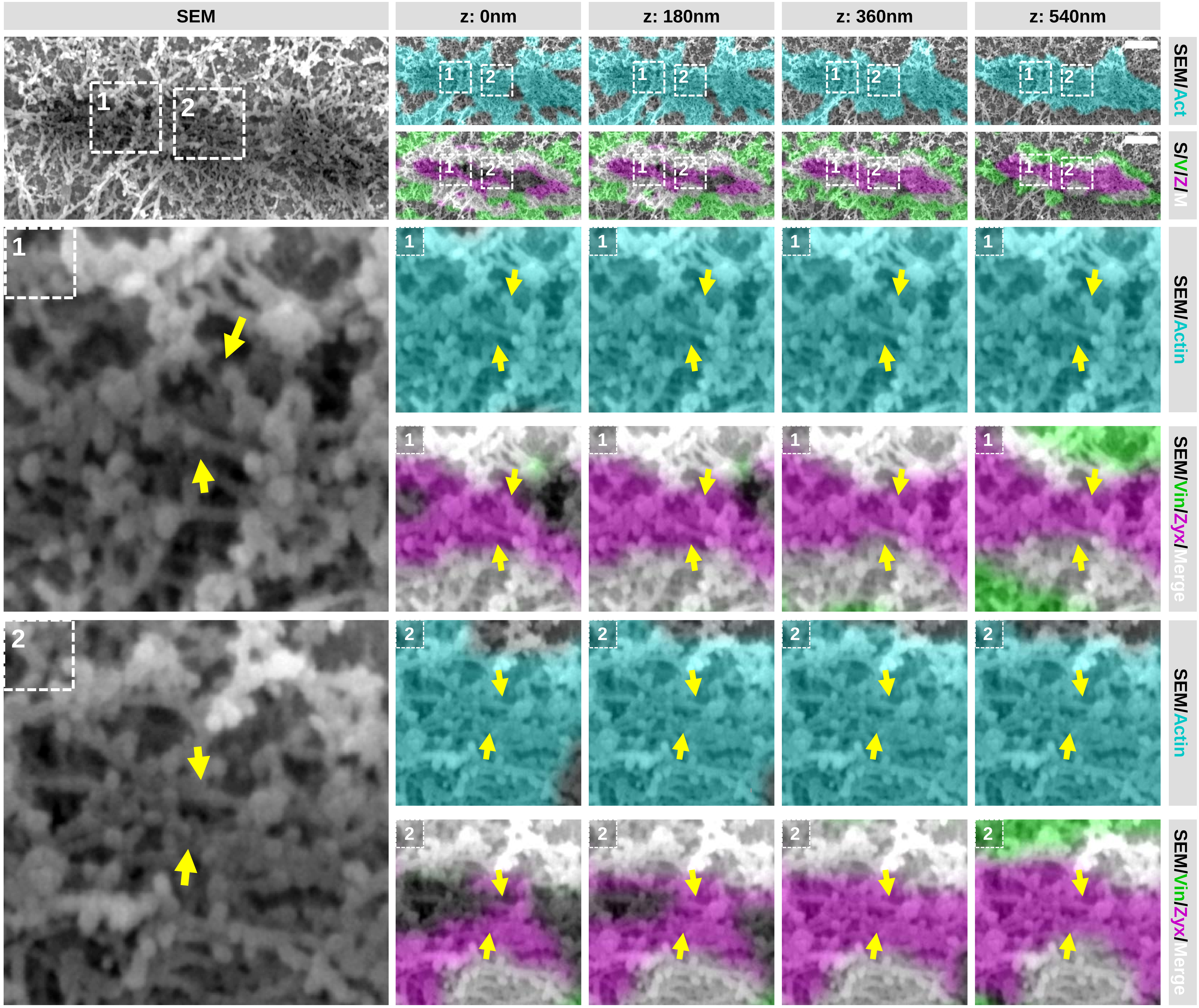

Supplementary Figure 9. Substrate topology induces interpodosomal connections

DCs were seeded on manually scratched glass coverslips and after VPM preparation, cells were fixed and stained for vinculin (green), zyxin (magenta) and actin (cyan). Overlay between vinculin and zyxin is shown in white. After CPD, DCs were imaged by SEM (gray). Shown is a representative area that contain multiple (at least 3) closely associated podosomes aligned on a topological feature that share one podosome ring (Figure 6 shows an additional example and corresponding individual LM channels are shown in Supplementary Figure 10). Arrows indicate the thick bundle of linear actin filaments that stretch from one core to the other. Scale bar = 500 nm.

Joosten et al. Supplementary Figure 10

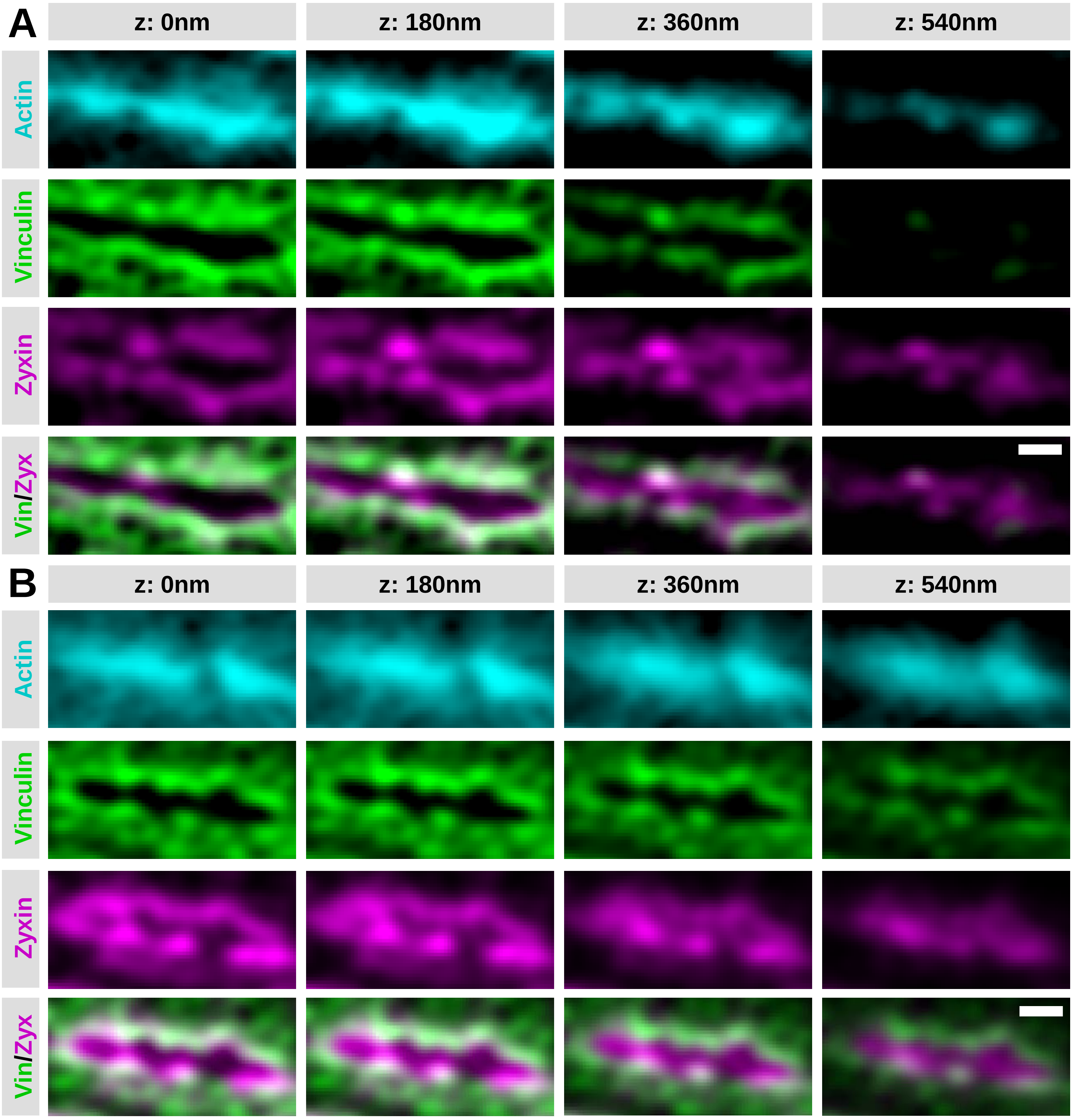

Supplementary Figure 10. Actin, vinculin and zyxin organization in closely associated podosomes on topographical cues
A-B) Shown are the individual LM images for actin (cyan), vinculin (green) and zyxin (magenta) as well as the vinculin/zyxin overlay corresponding to the podosomes shown in main Figure 6 (**A**) and Supplementary Figure 9 (**B**). Scale bar = 0.5 µm.

Joosten et al. Supplementary Figure 11

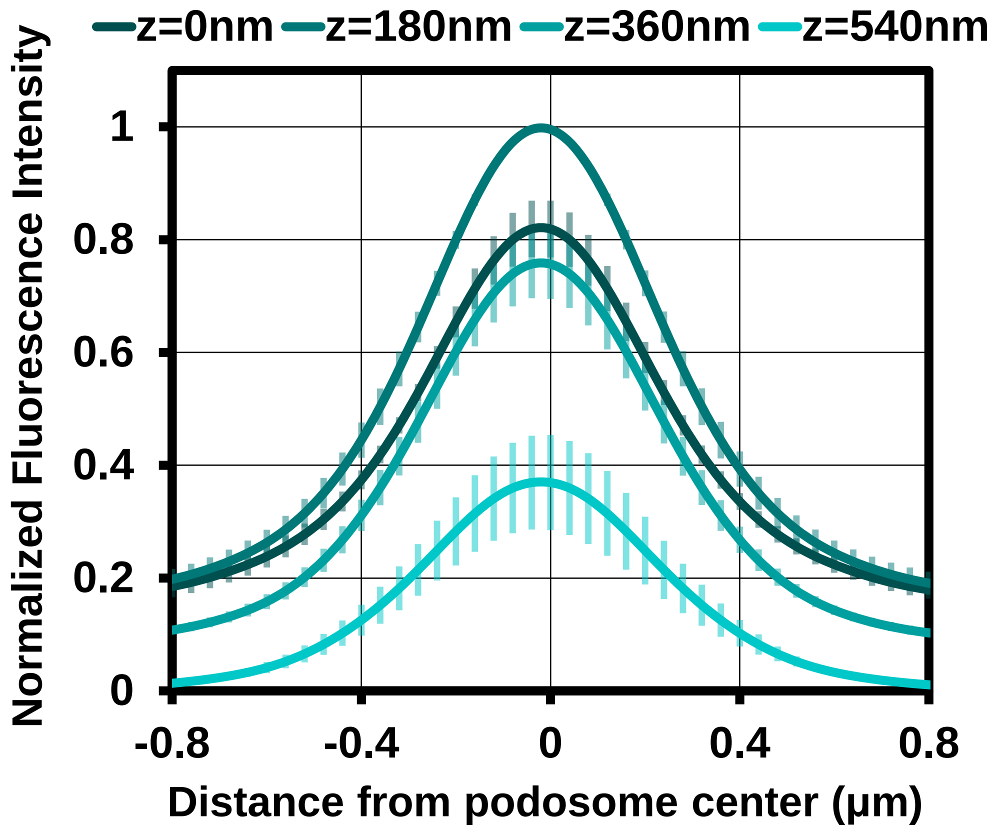

Supplementary Figure 11. Actin organization podosomes on topographical cues

Radial fluorescence profile analysis of actin at each of the four z-sections depicted in Figure 6. Shown is the average ± SEM (n = 140 podosomes, 4 cells).

Joosten et al. Supplementary Figure 12

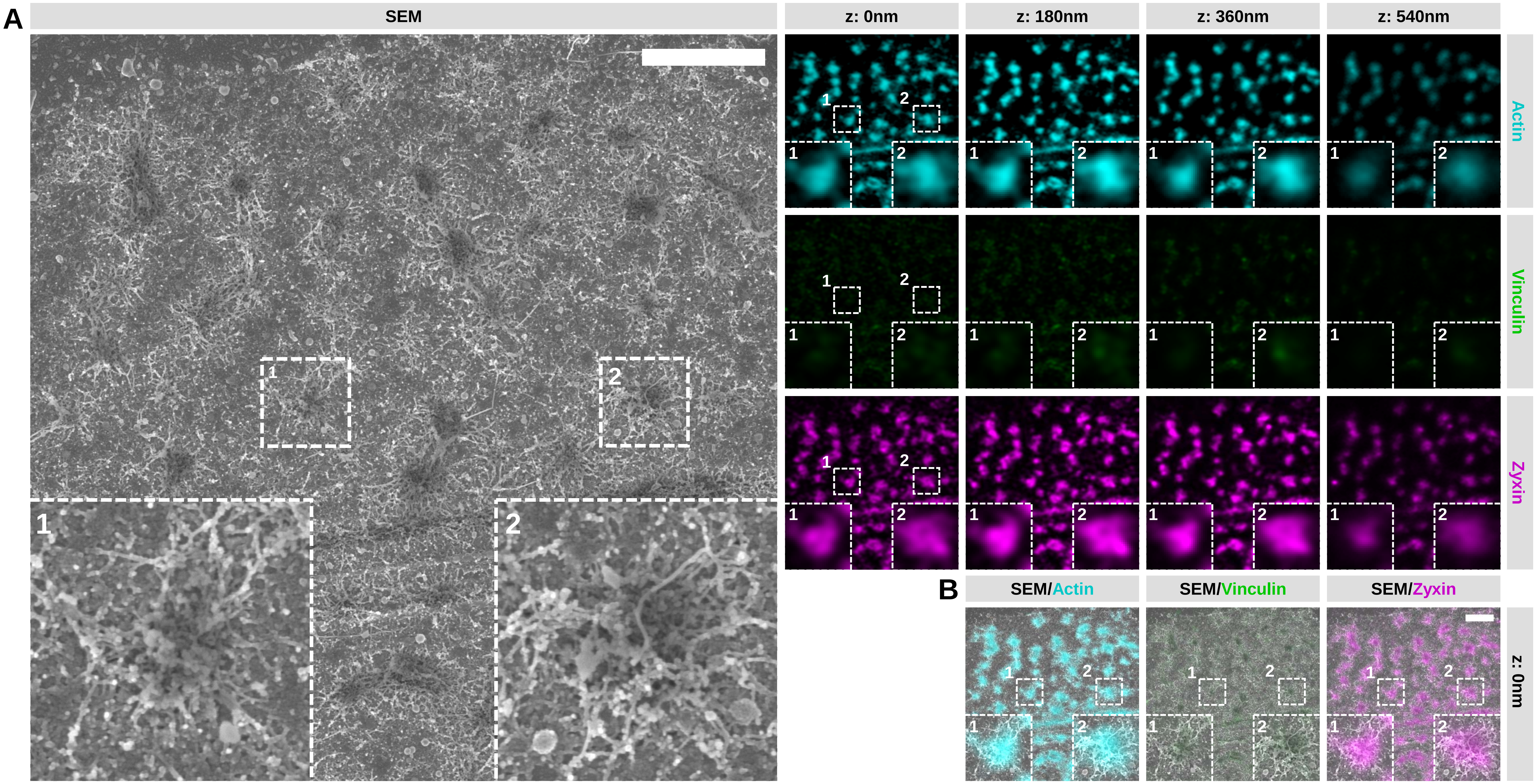

Supplementary Figure 12. Actin, vinculin and zyxin organization in podosome clusters in cells treated with CytoD

A) DCs were seeded on glass coverslips, treated for 10 minutes with CytoD and after VPM preparation, cells were fixed and stained for actin (cyan), vinculin (green) and zyxin (magenta). After CPD, DCs were imaged by SEM (gray). Shown are representative images of all three channels in 3 dimensions and the corresponding SEM image. Insets depict two representative podosomes within the cluster. **B)** Shown are the SEM-LM overlays for all three channels for the same cell as in **A**. Scale bar = 2 µm.

Joosten et al. Supplementary Figure 13

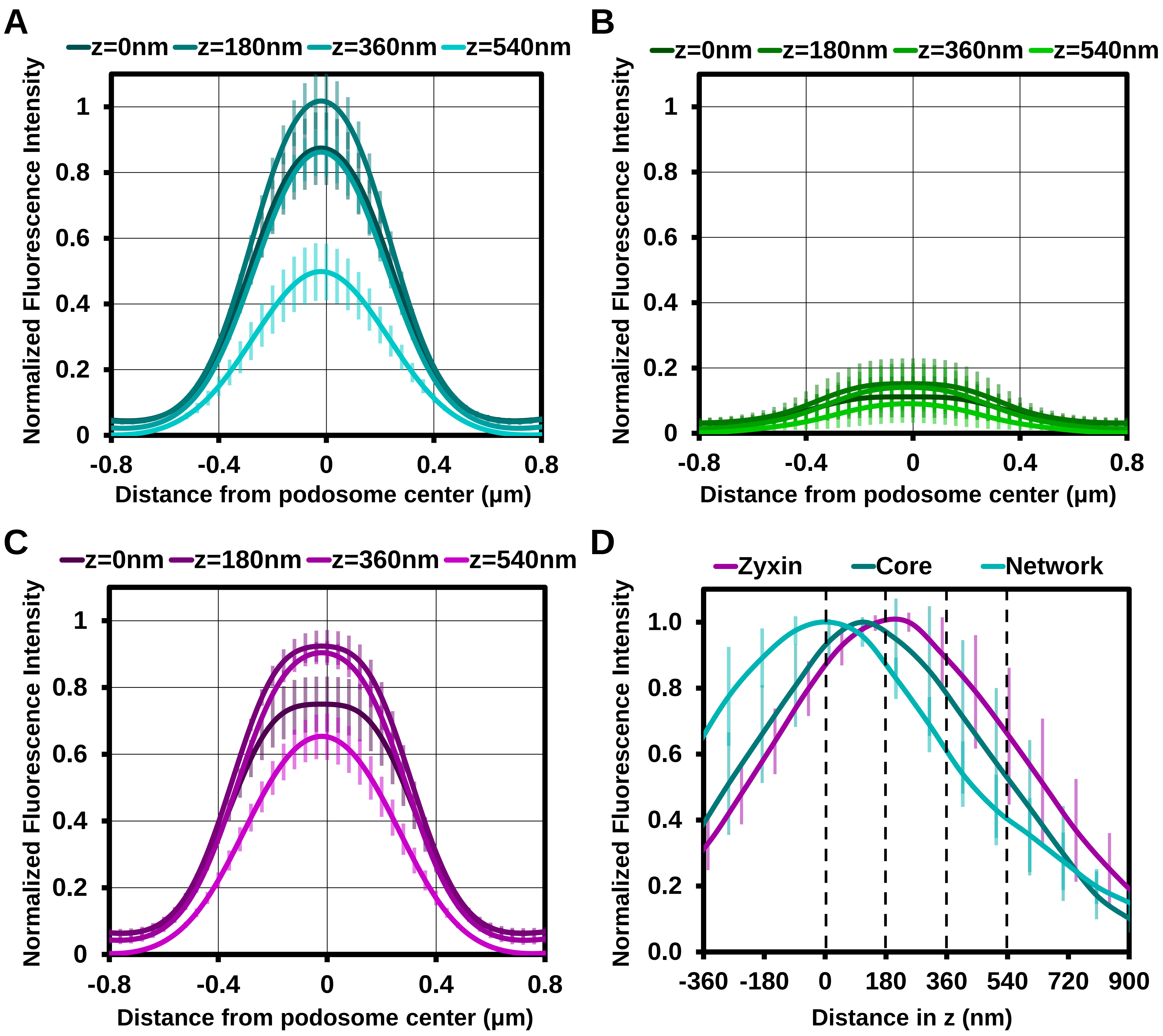

Supplementary Figure 13. Quantitative analysis of the organization of actin, vinculin and zyxin in podosome in cells treated with CytoD
A-C) Radial fluorescence profile analysis of actin (**A**), vinculin (**B**) and zyxin (**C**) of podosomes treated with CytoD at each of the four z-sections depicted in Supplementary Figure 12. Shown is the average ± SEM (n = 297 podosomes, 3 cells). **D)** Quantification of the localization in z of zyxin, core actin and network actin in podosomes treated with CytoD. The z-sections shown in Supplementary Figure 12 are represented by the dashed lines in the graph. Shown is the average ± SEM (n = 297 podosomes, 3 cells).

Joosten et al. Supplementary Figure 14

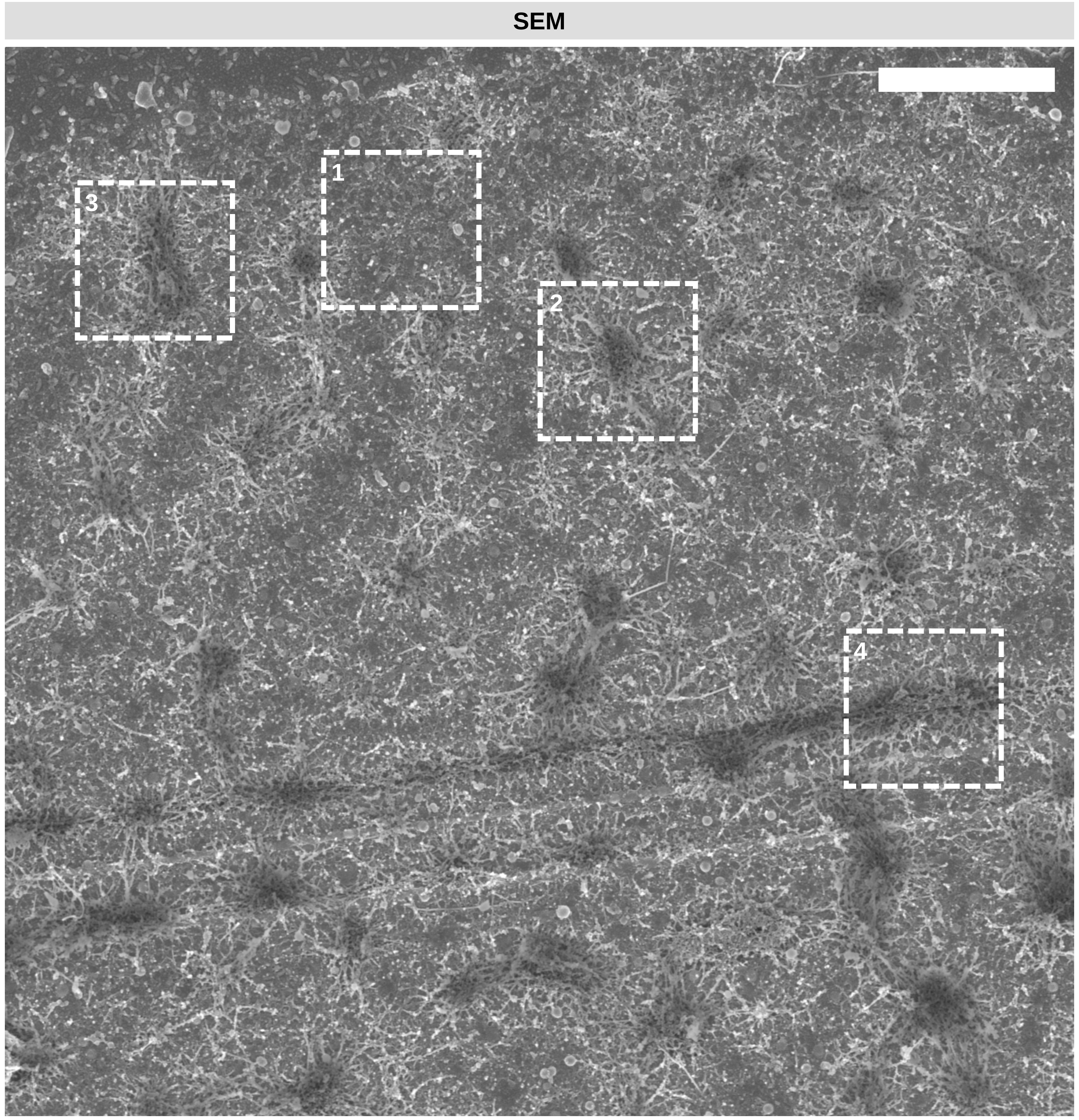

Supplementary Figure 14. Overview of the different areas in CytoD treated cell
Shown are the areas used for Figure 7 (1,2), Figure 8 (3) and Supplementary Figure 15 (4).

Joosten et al. Supplementary Figure 15

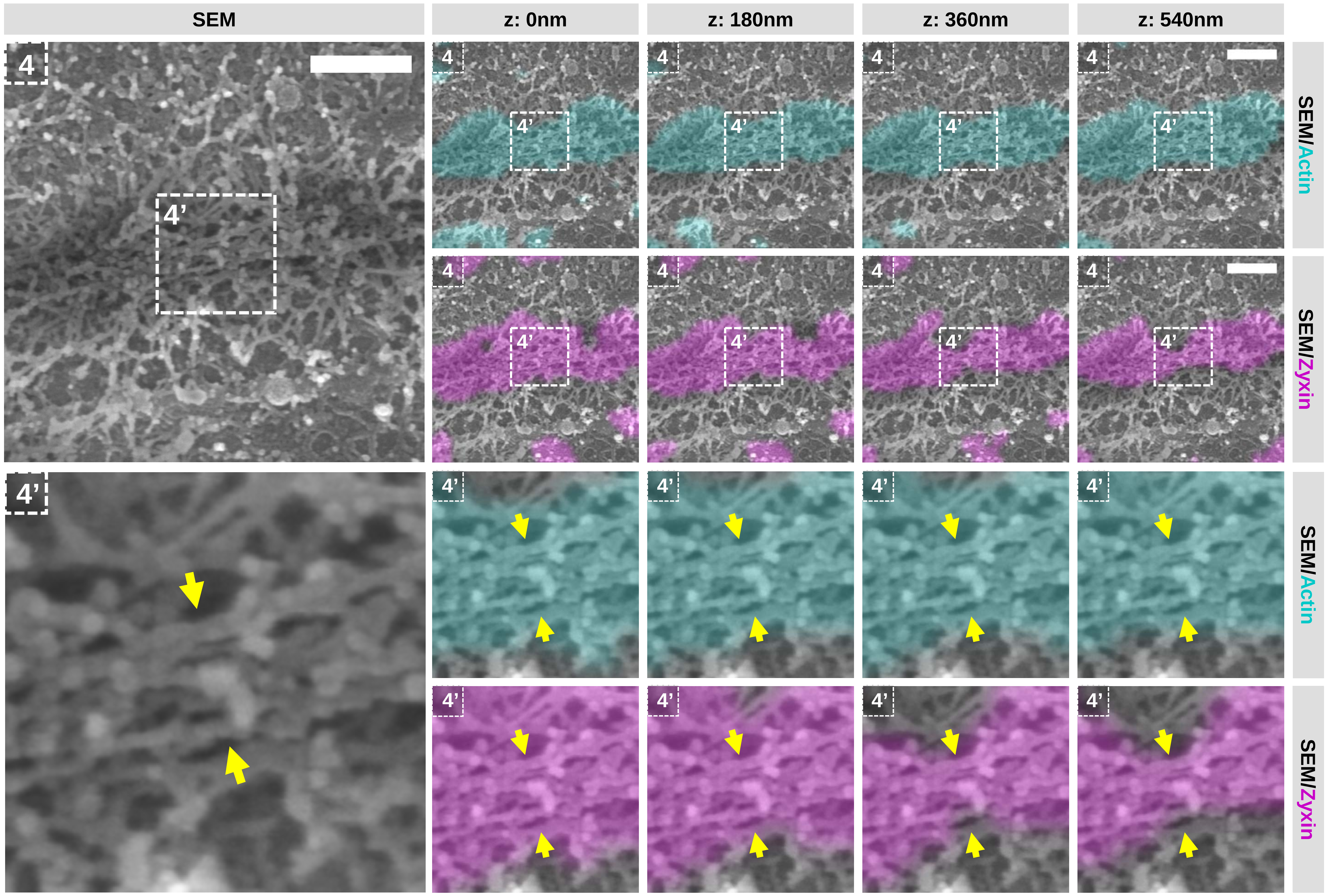

Supplementary Figure 15. Actin polymerization not essential for the integrity of the zyxin-associated interpodosomal connections
DCs were seeded on manually scratched glass coverslips, treated with CytoD for 10 minutes and after VPM preparation, cells were fixed and stained for actin (cyan), zyxin (magenta) and vinculin (not shown in this Figure). After CPD, DCs were imaged by SEM (gray). Shown is a representative event of closely associated podosomes on topographical cues (Supplementary Figure 14 shows the entire cell from which this event was selected and Supplementary Figure 16 shows the individual LM images). Arrows indicate the thick bundle of linear actin filaments that stretch from one core to the other. Scale bar = 400 nm.

Joosten et al. Supplementary Figure 16

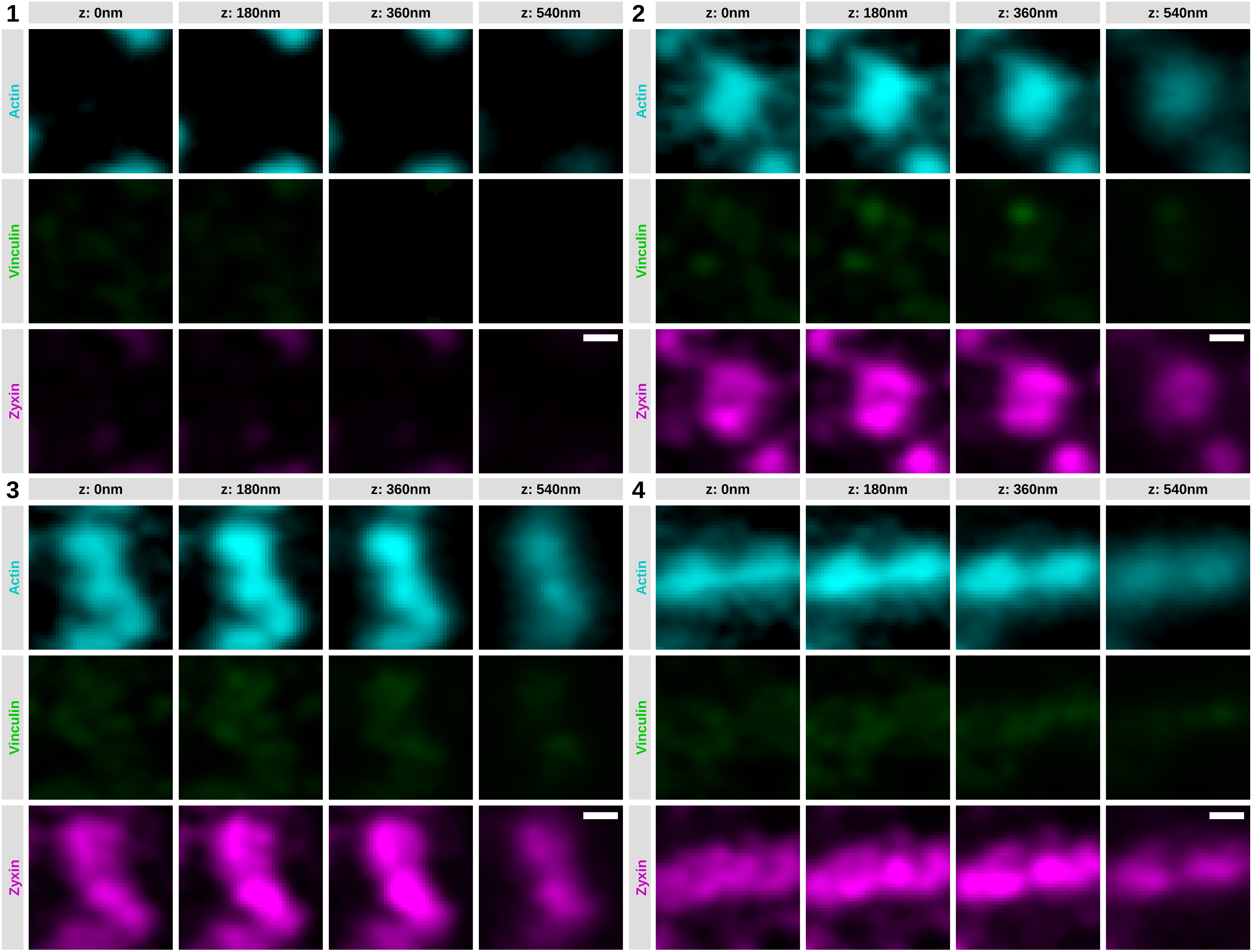

Supplementary Figure 16. Actin, vinculin and zyxin organization in podosomes treated with CytoD

1-4) Shown are the individual LM images for actin (cyan), vinculin (green) and zyxin (magenta) corresponding to the podosomes shown in main Figure 7 (1,2), Figure 8 (3) and Supplementary Figure 15 (4). Scale bar = 400 nm.
